# Myeloid malignancies with 5q and 7q deletions are associated with extreme genomic complexity, biallelic *TP53* variants, and very poor prognosis

**DOI:** 10.1038/s41408-021-00416-4

**Published:** 2021-02-08

**Authors:** Beth A. Pitel, Neeraj Sharma, Cinthya Zepeda-Mendoza, James B. Smadbeck, Kathryn E. Pearce, Joselle M. Cook, George Vasmatzis, Zohar Sachs, Rashmi Kanagal-Shamanna, David Viswanatha, Sheng Xiao, Robert B. Jenkins, Xinjie Xu, Nicole L. Hoppman, Rhett P. Ketterling, Jess F. Peterson, Patricia T. Greipp, Linda B. Baughn

**Affiliations:** 1 Department of Laboratory Medicine and Pathology, Division of Laboratory Genetics and Genomics, Mayo Clinic, Rochester, MN USA; 2 Center for Individualized Medicine-Biomarker Discovery, Mayo Clinic, Rochester, MN USA; 3 Department of Medicine, Division of Hematology, Mayo Clinic, Rochester, MN USA; 4 Division of Hematology, Oncology, and Transplantation, Department of Medicine and Masonic Cancer Center, University of Minnesota, Minneapolis, MN USA; 5 Department of Hematopathology, University of Texas MD Anderson Cancer Center, Houston, TX USA; 6 Department of Laboratory Medicine and Pathology, Division of Hematopathology, Mayo Clinic, Rochester, MN USA; 7 Department of Pathology, Brigham and Women’s Hospital, Harvard Medical School, Boston, MA USA; 8 Present Address: Cytogenetics and Genomic Microarray Laboratory, ARUP Laboratories, Salt Lake City, UT USA

**Keywords:** Cancer genomics, Cytogenetics, Cancer genomics, Cancer genetics, Cancer genetics

Dear Editor,

Acute myeloid leukemia (AML) is an aggressive myeloid neoplasm representing the most common type of acute leukemia in adults^1,2^. AML is classified into multiple genetic subtypes based on recurrent structural variations (SVs), copy number variations (CNVs), aneuploidies, and single nucleotide variants (SNVs). These genetic subtypes inform prognosis and influence clinical management^3–6^.

Cytogenetically visible deletions of chromosomes 5 and/or 7 in the absence of a WHO-defined recurrent SV represent a distinct subgroup associated with complex karyotype, pathogenic *TP53* variants, and adverse prognosis^4^. Identification of this subtype relies upon conventional cytogenetic techniques such as chromosome studies and/or fluorescence in situ hybridization (FISH)^4^. However, structural genomic complexity may be grossly underestimated and imprecise by these conventional chromosome studies and limited information is retrieved from FISH testing due to the targeted nature of the assay^7^. While genomic complexity has been explored using chromosomal microarray studies^8,9^, structural complexity profiling in AML by next-generation sequencing (NGS) has been largely unexplored. The objective of this study was to explore the relationship between 5q and 7q deletions, genome-wide genomic complexity as determined by NGS and conventional chromosome studies, *TP53* variants, and disease outcome.

## Study characteristics

**Fig. 1:**
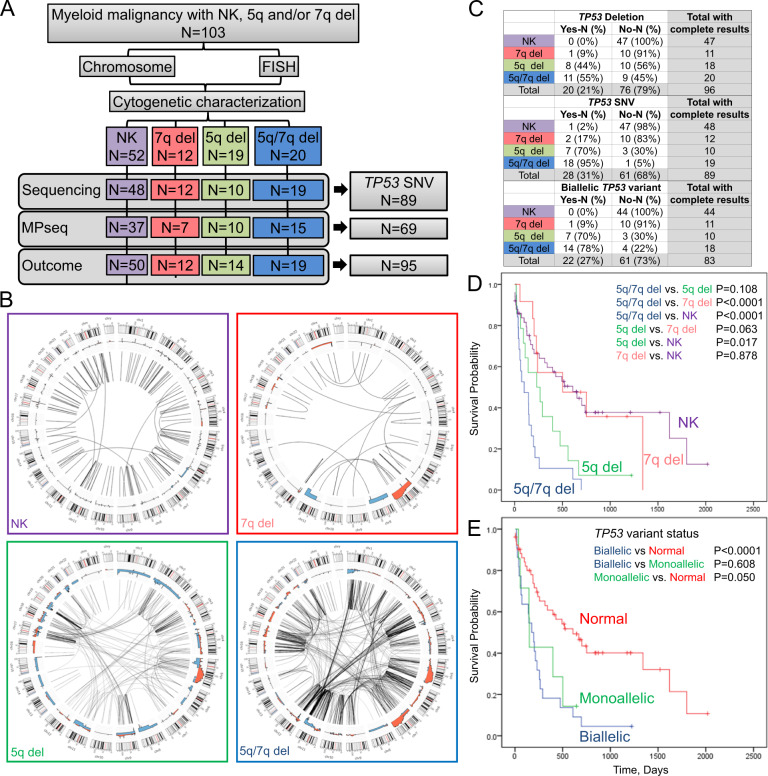
Myeloid clones with 5q and 7q deletions have complex genomes, biallelic pathogenic/likely pathogenic TP53 variants and poor overall survival. **A** Schematic of cohort. One hundred and three cases from patients with a diagnosis of a myeloid malignancy and with conventional chromosome and/or FISH studies demonstrating either normal karyotype (NK), 7q deletion (7q del), 5q deletion (5q del), 5q and 7q deletion (5q/7q del). Eighty-nine patients had sequencing (NGS or PCR-based) to identify sequence variants, 69 had MPseq to identify CNV and SV, and 95 samples had data available for assessment of overall survival. **B** Circos plots depicting CNVs and SVs detected by MPseq in each subtype. The outermost histogram (red) displays genomic losses, with axes rings representing the 20, 40, 60, 80, and 100% number of events per 1Mb window. The next histogram (blue) displays genomic gains, with axes rings representing the 20, 40, 60, 80, and 100% number of events per 1Mb window. Inner links (black) represent translocation and inversion events, with lines indicating positions along the chromosome. **C** *TP53* deletion determined by FISH or MPseq and single nucleotide variant (SNV) status. Biallelic status requires evidence of deletion and SNV, or two pathogenic/likely pathogenic SNVs or one SNV over 80% VAF. **D** Overall survival probability using NK, 5q and 7q patient status (*N* = 95 samples) and in **E**. *TP53* variant status (*N* = 81). Survival curves analysis was done using the Kaplan–Meier method and Log rank (Mantel–Cox) was run to determine the difference in the survival distribution among all four study subtypes. Eight patients were removed due to lack of follow-up data. Survival and AML diagnosis date was obtained from the medical record. The date of diagnosis reflects the original AML diagnosis. In **D**, 5q/7q del (blue line), 5q del (green line), 7q del (red line), and NK (purple line). In **E**, biallelic *TP53* variants (blue line), monoallelic *TP53* variants (green line), and normal *TP53* status (red line).

Following Mayo Clinic Institutional Review Board approval, we searched our Mayo Clinic Genomics database from 2017 and 2018 to identify patients that had a myeloid clone with a 5q deletion and/or 7q deletion identified by FISH and/or chromosomes studies (including cases with monosomies of chromosomes 5 and/or 7). We also identified cases with a normal karyotype (NK) AML clone as a control for low genomic complexity identified by conventional cytogenetics. A total of 103 cases had either a NK (NK, *N* = 52), 7q deletion (7q del, *N* = 12), 5q deletion (5q del, *N* = 19), or 5q deletion and 7q deletion (5q/7q del, *N* = 20) [Fig. 1A, Table S1].

The majority of cases, 90 (87%) represented diagnostic specimens and 13 (13%) were relapsed AML. Specifically, 48 (47%) were de novo AML, 37 (36%) were secondary AML (31 had AML with myeloid-related changes (MRC) and 6 were therapy-related). We also included five additional high-grade myeloid neoplasms with 5q del and/or 7q del including three patients with MDS (one with therapy-related MDS with 6% blasts, two with MDS with excess blasts; 12–20% blasts and 10–15% blasts) and two cases with an unspecified myeloid malignancy [Table S1, Table S2]. The median age was 68 years (range 9–90) with a slight male predominance of 53% [Table S1]. The European Leukemia Net (ELN) 2017 prognostication of patients in the NK subgroup depended largely on their SNVs^4^ [Table S1]. Nineteen (37%) NK samples could not be stratified due to incomplete sequencing data. Of 33 NK cases with ELN prognostication data, 10 were favorable, 11 were intermediate, and 12 were adverse. Of the remaining 51 cases that did not have NK, 48 cases had adverse risk due to identification of monosomy 5, 5q del, monosomy 7, complex karyotype and/or pathogenic/likely pathogenic variants (deletions or SNV) [Table S1]. While monosomy 7 is classified as high risk by ELN, 7q dels are classified as intermediate risk in the absence of other high-risk abnormalities. Two cases were classified as intermediate with a 7q del, a non-complex karyotype and no high-risk variants. One case had a 7q del, but evaluation for high-risk SNVs was incomplete [Table S1].

Sixty-nine cases had available DNA from bone marrow (BM) or peripheral blood (PB) for analysis by mate-pair sequencing (MPseq), a form of NGS optimized for the detection of SVs and CNVs^7^. Additional materials and methods details are in “Supplementary Information”. By MPseq, the minimum deleted region of chromosome 5q was ~6 Mb from 5q31.1 to 5q31.2 (chr5:134132000–139782000 [GRCh38]), encompassing *EGR1*, and the minimum deleted region of chromosome 7q was ~10 Mb from 7q32.1 to 7q34 (chr7:128933000–138962000 [GRCh38]) [Fig. 1B, Fig. S1]. No large deletions in the critical regions of chromosome 5q and 7q were identified by MPseq in NK samples [Fig. 1B, Fig. S1]. Genome-wide SVs and CNVs demonstrated overall increased genomic complexity of 5q del and 5q/7q del subtypes in comparison to NK and 7q del subtypes, with the greatest genomic complexity identified in the 5q/7q del subtype [Fig. 1B]. The median number of genome-wide CN gains, CN losses, and SVs were lower in NK (2.0, 5.0, and 4.0) and 7q del (2.0, 8.0, and 6.0) and higher in 5q del (11.5, 14.5, and 17.5) and 5q/7q del (14.0, 24.0, and 60.0), a difference that was significant among the 4 subtypes in each category (*p* < 0.001) [Table S3]. There was also an increased overall copy number burden (CNB) in cases with 5q/7q del, even when excluding any CN abnormalities involving 5q and 7q [Table S4]. Overall CNB correlated with karyotype complexity determined from the conventional chromosome results [Fig. S2]. Most 5q del and 5q/7q del subtypes were characterized by chromoplexy, chromothripsis, or progressive complexity with enrichment of SV involving chromosomes 5, 12, and 17, features absent in NK and 7q del cases [Fig. S3].

We next evaluated the incidence of pathogenic/likely pathogenic *TP53* variants (deletions and SNVs). *TP53* deletions were identified in 20/96 (21%) cases [Fig. 1C, Fig. S4]. None of the NK subtypes had a *TP53* deletion, 1 (9%) 7q del, 8 (44%) 5q del, and 11 (55%) 5q/7q del cases had a *TP53* deletion. Pathogenic/likely pathogenic *TP53* SNVs were identified in 28/89 (31%) cases. One NK case had a *TP53* SNV (~5% VAF), 2 (17%) 7q del, 7 (70%) 5q del, and 18 (95%) 5q/7q del had *TP53* SNVs. Monoallelic *TP53* variants were found in 7/83 (8%) cases and biallelic *TP53* variants were found in 22/83 (27%) of cases [Fig. 1C, Fig. S4]. Biallelic *TP53* variants were predominantly identified in cases with 5q del (70%) and 5q/7q del (78%). Fourteen of 16 cases (88%) with a *TP53* monoallelic deletion that were evaluable for *TP53* SNV had a *TP53* SNV. Two samples with a *TP53* deletion did not have a *TP53* SNV demonstrating that *TP53* deletion status is often predictive of a *TP53* SNV on the remaining allele. In contrast, 14 of 28 (50%) cases with a *TP53* SNV had a *TP53* deletion; the remaining 14 had a *TP53* SNV without a *TP53* deletion. No *TP53* pathogenic/likely pathogenic variants were identified in 5 complex karyotype-AML samples without 5q del and/or 7q del (data not shown). The type and location of each *TP53* SNV are shown in Fig. S5. Since *TP53* variants have been reported to associate with chromosome instability in myeloid cells^8,10,11^, cases with *TP53* SNVs had a higher median number of CN gains (14.0 vs. 2.0), CN losses (19.5 vs. 5.0), and SVs (51.0 vs. 4.0) compared to cases with normal *TP53*, with the greatest fold change (13-fold) was observed in the number of SVs in association with *TP53* variants [Fig. S5].

We next evaluated the contribution of 5q and 7q deletions, *TP53* variant status and genomic complexity on overall survival (OS). The median OS was significantly shorter for patients with 5q/7q del (100 days, 95%CI, 0–217 days, *p* < 0.0001) or 5q del (231 days, 95%CI, 2–460 days, *p* = 0.017) compared to NK (608 days, 95%CI, 300–918 days) and between 5q/7q del compared to 7q del (502 days, 95%CI, 0–1203 days) (*p* < 0.0001) [Fig. 1D] similar with prior reports^11,12^. No significant difference in OS was observed between 5q/7q del and 5q del and between NK and 7q del. The median OS was also significantly shorter for patients with biallelic (175 days, 95%CI, 102–247 days, *p* < 0.0001) or monoallelic *TP53* variants (150 days, 95%CI, 140–160 days, *p* = 0.050) compared to patients with normal *TP53* (608 days, 95%CI, 304–912 days). No significant difference in OS was observed between biallelic and monoallelic *TP53* categories, as previously reported^8^ (*p* = 0.608) [Fig. 1E]. Patients with high genomic complexity identified by MPseq and complex and monosomal karyotypes had a significantly shorter median OS compared to patients without these features (*p* < 0.0001) [Fig. S6]. The greatest risk of death was found in 5q/7q del (univariate risk ratio 3.39, *p* < 0.0001; 95%CI: 1.94–5.92 and multivariate risk ratio 2.58, *p* = 0.003; 95%CI: 1.36–4.88) in comparison to cases with only 5q del (univariate risk ratio 1.61, *p* = 0.124; 95%CI: 0.88–2.97). Cases with 7q del (univariate risk ratio 0.68, *p* = 0.306; 95%CI: 0.32–1.42) and NK (univariate risk ratio 0.46, *p* = 0.002; 95%CI: 0.28–0.75) had reduced risk of death compared to 5q/7q del [Table S5]. Improved OS of 7q del cases may be explained by 10 of 12 (83%) of 7q del cases had a simple karyotype, with <3 cytogenetic abnormalities, in contrast to 5q del or 5q/7q del cases, similar to previously published observations^11^. Similar OS between the 7q and NK cases may be due to the incorporation of NK cases with less favorable ELN risk categories based on mutation status (Table S1), further narrowing the OS gap between the NK and 7q del cohorts.

In summary, we describe the use of genome-wide NGS in the characterization of genomic complexity in AML, with the potential to reframe our understanding of complex genomic events. To our knowledge, very few studies have specifically evaluated the structural complexity incorporating both CNVs and SVs of AML genomes by NGS^13–15^. Here we show that myeloid malignancies with deletions of 5q and 7q are associated with additional complex genomic findings not appreciated by conventional chromosome studies including increased copy number burden, chromothripsis, chromoplexy, progressive genomic complexity, and very poor overall survival.

## Supplementary information

Supplemental Materials

Table S1

Table S2

Table S3

Table S4

Table S5

Figure S1

Figure S2

Figure S3

Figure S4

Figure S5

Figure S6
