# Supplementary material for: Myeloid malignancies with 5q and 7q deletions are associated with extreme genomic complexity, biallelic TP53 variants, and very poor prognosis
Source: Blood Cancer J. 2021 Feb 8;11(2):18. doi: 10.1038/s41408-021-00416-4 (PMC7873204; doi:10.1038/s41408-021-00416-4)
Supplement: Supplementary file 1 — Supplemental Materials [file 41408_2021_416_MOESM1_ESM.docx]

**SUPPLEMENTARY MATERIALS**

**Supplementary Methods**

**Data Collection and Ethical Considerations**

Data were collected on Mayo Clinic patients with FISH testing for AML and authorized research participation. SNV information was collected using the Mayo Clinic Genomics database. Medical history and survival information was accessed through EPIC electronic medical record system. Medical history queries in EPIC determined whether the instance of AML was considered de novo, therapy-related, or progression from other myeloid neoplasms. Survival was calculated from the date of diagnosis. All data are available upon request.

**Conventional G-banded chromosome studies**

Bone marrow cells were cultured, harvested and banded utilizing standard cytogenetic techniques as previously reported (1). Briefly, bone marrow was cultured without mitogens in PB-MAX media for 24 or 48 hours and treated with colcemid. Cells were harvested and fixed in an ethanol/glacial acetic acid solution. Slides were prepared and stained for G-banded karyotype analysis using standard GTL banding cytogenetic procedures. When available, 20 metaphases were analyzed and results reported per 2016 International System for Human Cytogenomic Nomenclature (ISCN).

**Fluorescence In-Situ Hybridization (FISH)**

Bone marrow aspirate specimens were harvested with or without prior culturing, and slides were prepared from the fixed cell suspension. Slides were pretreated using traditional cytogenetic methods and probes were hybridized to specific segments of DNA within the cells on the slide. DAPI was used to stain all nuclei. Recurrent abnormalities in AML were targeted for FISH analysis including inversions involving chromosomes 3 and 16; deletions of 5q, 7q, 13q, and 20q; gain of chromosome 8, rearrangements of *KMT2A* or *NUP98*, and several translocations: t(6;9)(*DEK*;*CAN*), t(8;21)(*RUNX1T1*;*RUNX1*), t(15;17)(*PML*;*RARA*), t(8;16)(*KAT6A*;*CREBBP*), t(9;22)(*ABL1*;*BCR*), and t(3;5)(*MLF1*;*NPM1*) (1).

**Single nucleotide variant (SNV) testing**

Approximately 200 ng of genomic DNA sheared to approximately 150 base-pairs was utilized for library preparation by the SureSelect XT library kit (Agilent, Santa Clara, CA) and sequenced on the HiSeq platform (Illumina, San Diego, CA) using 2x101 read lengths. NGS data was processed through a bioinformatics pipeline (Mayo NGS Workbench) for alignment, base calling and insertion/deletion (indel) detection. Results were confirmed by reviewing the BAM files in Alamut Visual (Interactive Biosoftware, Rouen, France) and SNVs were reported in GRCh37 (hg19). Biallelic *TP53* SNV was defined as the presence of a deletion and a pathogenic/likely pathogenic SNV, two pathogenic/likely pathogenic SNVs, or one pathogenic/likely pathogenic SNV with a variant allele frequency over 80% in the absence of a deletion. *TP53* sequencing targeted exons 4-10 of NM_000546.4. Sensitivity 5−10% variant allele fraction (VAF) with a minimum depth coverage of 250X for the NGS assay and the analytic sensitivity of the PCR assay is approximately 20%.

**DNA extraction and Mate Pair Library Preparation**

Genomic DNA was isolated using either the Qiagen Puregene extraction kit (for samples < 2 mL), Autopure LS Automated high quality DNA extraction (for samples >2 mL) or the QIAmp Tissue kit for fixed cell pellet samples. DNA was processed using the Illumina Nextera Mate Pair library preparation kit (Illumina) and sequenced on the Illumina HiSeq 2500 in rapid run mode as described in Aypar, et al. (1). Pooled libraries were hybridized onto a flow cell (2 samples per lane) and sequenced using 101-basepair reads and paired end sequencing.

**Structural Variant Bioinformatics Pipeline and Visualization**

The sequencing data was analyzed for the detection of structural variants (SVs), large genomic changes (>30Kb) involving junction breakpoints, and/or copy number variants (CNVs). Sequencing data were mapped to the reference genome (GRCh38) using BIMA (2) and the output was analyzed using SVAtools (1, 3, 4). Junctions and CNVs were graphically illustrated using genome, junction and region plots as previously described (1, 3, 5). Histograms for the number of occurrences of genomic gains and losses were calculated with BEDTools (6) by utilizing the number of events per 1Mb window along each human chromosome, build GRCh38 and visualized using Circos (7). Detection of SV and CNV by MPseq requires at least a 10% and 25% abnormal cell fraction in each sample, respectively (1).

**Structural complexity and copy number burden calculations**

To assess structural complexity, all junction plots from the SVs reported by SVAtools were manually evaluated for validity and tallied. Median values for each cohort were calculated. To assess copy number burden, the sum of the length of all copy number alterations in each sample was divided by the length of the autosomal GRCh38 genome using the coordinates per chromosome available in the UCSC genome browser: 2,875,001,522. These results were further stratified into burden related to copy number loss (CNLB) and copy number gain (CNGB).  CNB calculations were also performed excluding CNVs within the 5q and 7q regions and dividing by 2,642,549,290, the size of the autosomal GRCh38 genome excluding the 5q and 7q regions.

**Genomic Complexity Definitions**

Genomic complexity observed by MPseq was characterized as chromothripsis, chromoplexy, or progressive complexity as described (8-11). Chromosome junctions, representing genomic SVs, and gains/losses histograms were plotted using Circos (7). Chromoplexy was defined as ≥3 chromosomes involved in the same rearrangement. Chromothripsis was characterized as ≥5 deletions associated with the same rearrangement on either the same chromosome or two different chromosomes with alternating normal/deleted copy number. Progressive complexity was characterized as ≥5 CNVs per chromosome that was not defined as chromothripsis (11). Progressive complexity is also characterized by multiple copy number states and likely is derived from multiple chromosome shattering events (8, 10).

**Statistical analysis**

Statistical correlation for significance of the quantity of copy number gains, losses, and junctions was assessed by Kruskal Wallis test. The univariate and multivariate cox regression was done to estimate the survival risk of cytogenetic factors for overall survival. Statistical analyses performed using SPSS (IBM, Armonk, NY) and JMP (SAS Institute Inc., Cary, NC) with significance determined when *P* <0.05.

**References**

1. Aypar U, et al. Mate pair sequencing improves detection of genomic abnormalities in acute myeloid leukemia. Eur J Haematol. 2019;102(1):87-96.

2. Drucker TM, et al. BIMA V3: an aligner customized for mate pair library sequencing. Bioinformatics. 2014;30(11):1627-9.

3. Johnson SH, et al. SVAtools for junction detection of genome-wide chromosomal rearrangements by mate-pair sequencing (MPseq). Cancer Genet. 2018;221:1-18.

4. Smadbeck JB, et al. Copy number variant analysis using genome-wide mate-pair sequencing. Genes Chromosomes Cancer. 2018;57(9):459-70.

5. Gaitatzes A, Johnson SH, Smadbeck JB, Vasmatzis G. Genome U-Plot: a whole genome visualization. Bioinformatics. 2018;34(10):1629-34.

6. Quinlan AR, Hall IM. BEDTools: a flexible suite of utilities for comparing genomic features. Bioinformatics. 2010;26(6):841-2.

7. Krzywinski M, et al. Circos: an information aesthetic for comparative genomics. Genome Res. 2009;19(9):1639-45.

8. Korbel JO, Campbell PJ. Criteria for inference of chromothripsis in cancer genomes. Cell. 2013;152(6):1226-36.

9. Luijten MNH, Lee JXT, Crasta KC. Mutational game changer: Chromothripsis and its emerging relevance to cancer. Mutat Res. 2018;777:29-51.

10. Shen MM. Chromoplexy: a new category of complex rearrangements in the cancer genome. Cancer Cell. 2013;23(5):567-9.

11. Zhang CZ, Leibowitz ML, Pellman D. Chromothripsis and beyond: rapid genome evolution from complex chromosomal rearrangements. Genes Dev. 2013;27(23):2513-30.

**Supplementary Figure Legends**

**Figure S1:** **Plots depicting CNVs detected by MPseq in each subtype for chromosomes 5 and 7.** Focus visualization of chromosomes 5 and 7 with minimal copy region of overlap indicated as * for chromosome 5 and ** for chromosome 7. The outermost histogram (red) displays genomic losses, with axes rings representing the 20%, 40%, 60%, 80%, and 100% number of events per 1Mb window. The next histogram (blue) displays genomic gains, with axes rings representing the 20%, 40%, 60%, 80%, and 100% number of events per 1Mb window.

**Figure S2: Copy Number Burden (CNB) in relation to karyotype complexity.**  CNB, CNLB, CNGB (including 5q/7q chromosome regions) and number of SVs from Table S4 in relation to chromosome complexity obtained from the conventional chromosome study from Table S2.

**Figure S3: Presence of chromoplexy and chromothrypisis/progressive complexity in each subtype. A.** Chromoplexy (top, green boxes), chromothripsis (orange boxes) and progressive complexity (red boxes) (bottom) depicted in each subtype. The number in the box refers to the number of SVs identified in each rearrangement. The yellow box indicates chromosome 20 was involved in 2 separate rearrangements between chromosomes 4,5,10,12 17 and 20 as well as 6, 20 and 21. The number of cases involving each chromosome is indicated as “# of cases”. No chromoplexy, chromothripsis or progressive complexity was observed for 7q del and NK subtypes. Six of 10 (60%) 5q del and 11/15 (73%) 5q/7q del subtypes displayed evidence of chromoplexy. The most common chromosomes involved in chromoplexy included chromosomes 5, 7, 12, and 17 and no chromoplexy rearrangements were identified on chromosomes 13, 15, X and Y. Six of 10 (60%) of 5q cases and 14/15 (93%) 5q/7q del displayed evidence of either chromothripsis or progressive complexity. Chromosomes 5, 12 and 17 were most often associated with chromoplexy, chromothripsis or progressive complexity, and events of structural complexity on chromosome 12 were often related to CNVs or SVs of *ETV6*. **B.** Recurrent regions of CNV or SV appeared enriched in the 5q/7q del subtype including terminal 1p gains (33%), *MECOM* rearrangements (20%), *ETV6* CNVs or SVs (33%), terminal 11q gains (47%) and 20q deletions (40%). The 1p duplication ranged in size from 3,741 kb (1p36.33p36.32) to 91,202 kb (1p36.33p22.2). **T**he region of common overlap of 1p included *PRDM16* (1p36.32). **Additionally, 3 cases had a very similar 11q terminal gain footprint arising from severe progressive complexity. The region of gain ranged in size from 16,149 kb to 20,576 kb and included the *CBL*, *FLI1*, *PRDM10*, and *ARHGEF12* genes, with *KMT2A* located 457 kb proximal to the minimal region of complex gain.**

**Figure S4: *TP53* variants in each subtype and in relation to genomic complexity. A.** *TP53* deletion and single nucleotide variant (SNV) status: No pathogenic/likely pathogenic *TP53* variant is indicated by an “N” in the status column and a white box in the deletion and SNV columns. Unknown *TP53* status is indicated by a “U” in the status column and a grey box in the deletion and SNV columns. Biallelic *TP53* variation is indicated by a “B” in the status column and a maroon box in the deletion and SNV columns. Monoallelic *TP53* variation is indicated by an “M” in the status box and a red box in the deletion and SNV columns. If a maroon or red box is observed in the deletion column, there is a biallelic or monoallelic deletion, respectively. If a maroon or red box is observed in the SNV column, there is a biallelic or monoallelic SNV, respectively. Biallelic status requires evidence of a deletion and SNV, or two pathogenic/likely pathogenic SNVs or one SNV over 80% VAF. 5q/7q-98 has unknown biallelic status. NK-15 has an Arg290His VUS at 49% VAF that was not included in the figure because it was not considered a pathogenic or likely pathogenic variant. **B.** Of cases with MPseq and complete *TP53* variant status data available, median number of losses, gains and SVs were calculated in cases with normal *TP53* status compared to cases with either a *TP53* deletion or SNV.

**Figure S5: Type and location of *TP53* SNVs.** Protein position and domain location was determined using the St. Jude Pecan portal. No pathogenic/likely pathogenic *TP53* variant is indicated by an “N” in the status column and a white box in the deletion and SNV columns. Unknown *TP53* status is indicated by a “U” in the status column and a grey box in the deletion and SNV columns. Biallelic *TP53* variation is indicated by a “B” in the status column and a maroon box in the deletion and SNV columns. Monoallelic *TP53* variation is indicated by an “M” in the status box and a red box in the deletion and SNV columns. If a maroon or red box is observed in the deletion column, there is a biallelic or monoallelic deletion, respectively. If a maroon or red box is observed in the SNV column, there is a biallelic or monoallelic SNV, respectively. Biallelic status requires evidence of a deletion and SNV, or two pathogenic/likely pathogenic SNVs or one SNV over 80% VAF. 5q/7q-98 has unknown biallelic status.

**Figure S6: Overall survival between presence or absence of genomic complexity identified by MPseq or chromosome studies.** Survival curve analysis was done using the Kaplan-Meier method and Log rank (Mantel-Cox) was run to determine the difference in the survival distribution among the variables. Evidence of chromothripsis/progressive complexity and chromoplexy determined using data from Fig. S3. Chromosome identified abnormalities (complexity and monosomal karyotype) were derived from Table S2. Monosomal karyotype is defined by cases with at least 2 autosomal monosomies or an autosomal monosomy and with at least one structural abnormality by conventional chromosome studies.
