# Supplementary material for: Myeloid malignancies with 5q and 7q deletions are associated with extreme genomic complexity, biallelic TP53 variants, and very poor prognosis
Source: Blood Cancer J. 2021 Feb 8;11(2):18. doi: 10.1038/s41408-021-00416-4 (PMC7873204; doi:10.1038/s41408-021-00416-4)
Supplement: Supplementary file 2 — Table S1 [file 41408_2021_416_MOESM2_ESM.docx]

**Table S1: Study group characteristics**

| **Characteristics** | **NK  (N=52)** | **7q del  (N=12)** | **5q del  (N=19)** | **5q/7q del  (N=20)** | **Total (N=103)** |
| --- | --- | --- | --- | --- | --- |
|  |  | **(N=51)** | | |  |
| **Gender (male)** | 29 (56%) | 4 (33%) | 8 (42%) | 14 (70%) | 55 (53%) |
| **Median age (years)** | 69 | 67 | 68 | 70 | 68 |
| **Diagnosis** |  |  |  |  |  |
| **De novo AML** | 28 (54%) | 4 (33%) | 6 (32%) | 10 (50%) | 48 (47%) |
| **Secondary AML: AML-MRC** | 15 (29%) | 5 (42%) | 7 (37%) | 4 (20%) | 31 (30%) |
| **Secondary AML: Therapy related** | 1 (2%) | 1 (8%) | 2 (11%) | 2 (10%) | 6 (6.0%) |
| **MDS/other myeloid malignancy** | 0 (0%) | 0 (0%) | 2 (11%) | 3 (15%) | 5 (5.0%) |
| **Relapsed AML** | 8 (15%) | 2 (17%) | 2 (11%) | 1 (5%) | 13 (13%) |
| **ELN risk** |  |  |  |  |  |
| **Favorable** | 10 (19%) | 0 | 0 | 0 | 10 (10%) |
| **Intermediate** | 11 (21%) | 2 (17%) | 0 | 0 | 13 (13%) |
| **Adverse** | 12 (23%) | 9 (75%) | 19 (100%) | 20 (100%) | 60 (58%) |
| **Unknown** | 19 (37%) | 1 (8%) | 0 | 0 | 20 (19%) |
| **Somatic variants** |  |  |  |  |  |
| ***NPM1* variant, normal *FLT3* ITD or *FLT3* ITD low** | 5/36 (14%) | 0/5 | 0/4 | 0/12 | 5/57 (9%) |
| ***CEBPA* biallelic variant** | 5/36 (14%) | 0/5 | 0/4 | 0/12 | 5/57 (9%) |
| ***NPM1* variant*, FLT3* ITD high** | 2/36 (6%) | 0/5 | 0/4 | 0/12 | 2/57 (4%) |
| **Normal *NPM1,* normal *FLT3* ITD or *FLT3* ITD low, normal *ASXL1*, *RUNX1* or *TP53*** | 9/29 (31%) | 0/5 | 0/4 | 0/12 | 9/50 (18%) |
| **Normal *NPM1, FLT3* ITD high** | 3/36 (8%) | 0/5 | 0/4 | 0/12 | 3/57 (5%) |
| ***ASXL1* and/or *RUNX1* variant** | 9/29 (31%) | 2/5 (40%) | 1/4 (25%) | 1/10 (10%) | 13/48 (27%) |
| ***TP53* biallelic variant** | 0/44 | 1/11 (9%) | 7/10 (70%) | 14/18 (78%) | 22/83 (27%) |

Patient characteristics: 103 samples with patient sex, age and morphologic diagnosis at time of genomic testing, and SNV profile in relation to cytogenetic subtypes. ELN risk stratification was used to determine adverse, intermediate, or favorable risk status*. The number of cases with somatic pathogenic or likely pathogenic variants are recorded as a proportion of the total number of cases evaluated for each variant tested.

*Dohner H, et al. Diagnosis and management of AML in adults: 2017 ELN recommendations from an international expert panel. Blood. 2017;129(4):424-47.
