# Supplementary material for: Myeloid malignancies with 5q and 7q deletions are associated with extreme genomic complexity, biallelic TP53 variants, and very poor prognosis
Source: Blood Cancer J. 2021 Feb 8;11(2):18. doi: 10.1038/s41408-021-00416-4 (PMC7873204; doi:10.1038/s41408-021-00416-4)
Supplement: Supplementary file 3 — Table S2 [file 41408_2021_416_MOESM3_ESM.docx]

**Table S2: Patient characteristics**

| **Case ID** | **Sex** | **Age** | **Disease Classification** | **Karyotype** | **Complex #** |
| --- | --- | --- | --- | --- | --- |
| NK-1 | F | 68 | De novo AML | ND, FISH normal | N/A |
| NK-2 | M | 52 | Relapsed AML | 46,XY[8]* | 0 |
| NK-3 | M | 74 | De novo AML | 46,XY[20] | 0 |
| NK-4 | M | 78 | AML-MRC | 46,XY[20] | 0 |
| NK-5 | M | 55 | De novo AML | 46,XY[20] | 0 |
| NK-6 | F | 58 | De novo AML | 46,XX[20] | 0 |
| NK-7 | F | 77 | AML-MRC | 46,XX[20] | 0 |
| NK-8 | F | 9 | De novo AML | 46,XX[20] | 0 |
| NK-9 | F | 34 | De novo AML | 46,XX[20] | 0 |
| NK-10 | M | 78 | De novo AML | 46,XY[20] | 0 |
| NK-11 | M | 71 | Relapsed AML | 46,XY[20] | 0 |
| NK-12 | F | 84 | Relapsed AML | 46,XX[20] | 0 |
| NK-13 | F | 65 | AML-MRC | 46,XX[20] | 0 |
| NK-14 | F | 72 | AML-MRC | ND, FISH normal | N/A |
| NK-15 | F | 68 | AML-MRC | 46,XX[20] | 0 |
| NK-16 | M | 68 | De novo AML | 46,XY[20] | 0 |
| NK-17 | M | 73 | De novo AML | 46,XY[20] | 0 |
| NK-18 | F | 71 | AML-MRC | 46,XX[20] | 0 |
| NK-19 | F | 39 | De novo AML | 46,XX[20] | 0 |
| NK-20 | M | 51 | Relapsed AML | 46,XY[18]* | 0 |
| NK-21 | M | 90 | Therapy related AML | 46,XY[20] | 0 |
| NK-22 | M | 65 | De novo AML | 46,XY[20] | 0 |
| NK-23 | M | 80 | De novo AML | 46,XY[20] | 0 |
| NK-24 | M | 84 | AML-MRC | 46,XY[20] | 0 |
| NK-25 | M | 30 | De novo AML | 46,XY[20] | 0 |
| NK-26 | M | 79 | De novo AML | 46,XY[20] | 0 |
| NK-27 | F | 74 | De novo AML | 46,XX[15] | 0 |
| NK-28 | F | 71 | De novo AML | 46,XX[20] | 0 |
| NK-29 | M | 70 | De novo AML | 46,XY[20] | 0 |
| NK-30 | M | 65 | AML-MRC | 46,XY[20] | 0 |
| NK-31 | M | 64 | De novo AML | 46,XY[20] | 0 |
| NK-32 | F | 38 | Relapsed AML | ND, FISH normal | N/A |
| NK-33 | F | 66 | De novo AML | 46,XX[20] | 0 |
| NK-34 | F | 74 | Relapsed AML | 46,XX[20] | 0 |
| NK-35 | M | 38 | De novo AML | 45,X,-Y[11]/46,XY[9] | 1 |
| NK-36 | F | 53 | De novo AML | 46,XX[20] | 0 |
| NK-37 | M | 28 | De novo AML | 46,XY[20] | 0 |
| NK-38 | M | 18 | Relapsed AML | 46,XY[20] | 0 |
| NK-39 | F | 66 | AML-MRC | 46,XX[20] | 0 |
| NK-40 | F | 59 | De novo AML | 46,XX[20] | 0 |
| NK-41 | M | 87 | De novo AML | ND, FISH normal | N/A |
| NK-42 | M | 87 | De novo AML | 46,XY[20] | 0 |
| NK-43 | M | 77 | AML-MRC | 46,XY[20] | 0 |
| NK-44 | M | 69 | De novo AML | 46,XY[20] | 0 |
| NK-45 | M | 51 | De novo AML | 46,XY[20] | 0 |
| NK-46 | F | 67 | AML-MRC | 46,XX[20] | 0 |
| NK-47 | F | 77 | AML-MRC | 46,XX[20] | 0 |
| NK-48 | M | 20 | Relapsed AML | 46,XY[20] | 0 |
| NK-49 | M | 81 | AML-MRC | 46,XY[20] | 0 |
| NK-50 | M | 77 | De novo AML | 46,XY[20] | 0 |
| NK-51 | F | 88 | AML-MRC | 46,XX[20] | 0 |
| NK-52 | F | 83 | AML-MRC | 46,XX[20] | 0 |
| 7q-53 | F | 67 | AML-MRC | 44,XX,**-7**,-17,der(18)t(17;18)(q11.2;q11.2)[18]/46,XX[2] | 4 |
| 7q-54 | M | 74 | AML-MRC | 45,XY,**-7[7]**/45,idem,del(12)(p11.2p13)[13] | 2 |
| 7q-55 | F | 58 | Therapy related AML | 46,XX,**der(7)t(7;11)(q11.2;q13)[20]** | 2 |
| 7q-56 | F | 38 | Relapsed AML | 46,XX,add(3)(q27),**del(7)(q22)**,add(12)(p11.2)[cp18]/46,idem,t(1;2)(q21;p13)[2] | 4 |
| 7q-57 | F | 82 | AML-MRC | 45,XX,**-7[18**]/46,sl,+8[2] | 2 |
| 7q-58 | M | 66 | AML-MRC | 45,XY,**-7[12]**/47,XY,+8[1]/46,XY[7] | 2 |
| 7q-59 | F | 55 | Relapsed AML | 46,XX,**del(7)(q21q36)[11]**//46,XY[9] | 1 |
| 7q-60 | F | 53 | De novo AML | 45,XX,**-7[12]**/46,XX[8] | 1 |
| 7q-61 | M | 68 | De novo AML | 46,XY,**der(7)t(7;15)(q22;q21)[5]**/46,XY[15] | 2 |
| 7q-62 | F | 70 | De novo AML | 45,XX,-**7[1]**/45,idem,add(3)(q27)[19] | 2 |
| 7q-63 | F | 51 | De novo AML | 46,XX,**idic(7)(q11.2)[7]**/46,XX[13] | 1 |
| 7q-64 | M | 74 | AML-MRC | 45,XY,**-7[8]**/46,idem,+r[3]/46,XY[9] | 2 |
| 5q-65 | F | 72 | AML-MRC | 45,XX,add(4)(q21),**add(5)(q11.2)**,add(7)(p13),der(16)t(16;18)(q13;q11.2),-18,add(21)(q22)[14]/46,XX,t(11;17)(q23;q11.2)[6] | 7 |
| 5q-66 | M | 56 | Relapsed AML | 44,X,-Y,**i(5)(p10)**, -16[2]/44,idem,der(4)t(4;16)(q13;q22),der(6)t(4;6)(q13;q23)[8]//46,XX[10] | 3 |
| 5q-67 | M | 72 | De novo AML | 44-45,XY,+der(4)add(4)(p12)t(4;6)(q35;p21.3),der(4)add(4)(p12)t(4;6)(q35;p21.3),**-5**,-6,+add(8)(p23),-11,add(11)(p13),-17, add(18)(q21),del(20)(q11.2q13.3),+0-mar[cp20] | 11 |
| 5q-68 | M | 73 | AML-MRC | 56-65,XX,+X,+1,+der(2)add(2)(p11.2)add(2)(q35),add(3)(q21),+5,**del(5)(q13q33)x2**,+6,+6,+8,+8,+9,+der(11)t(11;12)(p13;q15),add(12)(q24.3),+13,+14,+15,+16,+18,+add(18)(q23),+20,add(21)(p11.2),+22,add(22)(p11.2),+1-3mar[cp20] | 26 |
| 5q-69 | M | 59 | MDS/other myeloid malignancy | 44-46,XY,del(3)(p13p21),**-5**,add(9)(q13),add(12)(q13),-18,-22,+0-2r,+1-2mar[cp16]/88-93,idemx2[cp3]/46,XY[1] | 11 |
| 5q-70 | F | 67 | AML-MRC | 54,XX,+1,del(4)(q21q27),+5,**del(5)(q13q33)x2**,+8,t(10;22)(p15;q11.2),+der(10)t(10;22),+11,+15,add(17)(p13),+18,+21[15]/46,XX[5] | 14 |
| 5q-71 | F | 30 | De novo AML | 46,XX,**del(5)(q31q33)**,add(11)(p15),add(18)(q21)[16]/46,sl,add(6)(q21)[2]/46,XX[2] | 4 |
| 5q-72 | F | 68 | MDS/other myeloid malignancy | 45,XX,add(1)(p13),der(2)t(1;2)(p13;q11.2),-3,**der(5)t(2;5)(q13;q13)**,add(7)(p11.1),-9,-9,-17,-19,+4mar[19]/46,XX[1] | 15 |
| 5q-73 | F | 69 | De novo AML | 47-51,XX,**der(5)ins(5)(p13)t(5;18)(q11.2;q11.2)**,der(15)t(8;15)(q13;q26.1),-17,-18,+21,i(21)(q10),+der(?)t(?;17)(?;q11.2),+4-9mar[cp20] | 19 |
| 5q-74 | F | 60 | Therapy related AML | 43,XX,add(2)(p23),-4,**der(5)t(5;14;19)(q34;q32;q13.2)del(5)(q14q34),**  i(11)(q10),der(12)del(12)(p11.2)?dup(12)(q14q23),der(14)t(5;14;19),-16,-17,  der(19)t(5;14;19),del(20)(q11.2)[cp20] | 18 |
| 5q-75 | M | 60 | Therapy related AML | 41-43,X,-Y,-5,**add(5)(q11.2),**add(7)(p11.2),-10,add(10)(q22),-13,-14,-16,add(16)(q13),-17,-18,add(19)(p13.3),-20,+0-4mar[cp15]/46,XY[5]. **No 7q del by FISH** | 18 |
| 5q-76 | F | 61 | Relapsed AML | 48,XX,+13,del(17)(p11.2),+19[10] **FISH confirms 5q del** | 3 |
| 5q-77 | F | 70 | De novo AML | 46,XX,**t(5;6)(q33;q22),**del(17)(p11.2)[14]/46,XX[6] | 2 |
| 5q-78 | M | 58 | AML-MRC | 45,XY,add(2)(p25),**add(5)(q11.2)**,del(9)(q13q22),del(16)(q22),der(17)add(17)(p11.2)add(17)(q21),-21,-21,+mar[14]/46,XY[6] | 8 |
| 5q-79 | M | 66 | AML-MRC | 46,XY,**del(5)(q13q33)**,del(11)(p11.2p13),del(20)(q11.2q13.3)[20] | 3 |
| 5q-80 | F | 74 | AML-MRC | 46,XX,del(20)(q11.2q13.3)[11]/46,sl,**del(5)(q31q35)[7]**/46,XX[2] | 2 |
| 5q-81 | F | 70 | AML-MRC | 46,XX,add(3)(p13),-**5**,add(6)(q21),+8,add(12)(p11.2),add(12)(q24.1),add(18)(p11.2)[14]/46,sl,del(16)(q12)[2]/46,sl,der(19)t(11;19)(q13;p13.3)[2]/47,sl,+21[2] | 11 |
| 5q-82 | F | 78 | De novo AML | 46,XX,**del(5)(q13q33)**[1]/46,sl,del(3)(q23)[13]/45,sdl1,add(21)(p11.2)[3]/46,XX[3] | 3 |
| 5q-83 | M | 70 | De novo AML | 44-45,XY,t(3;9)(p21;q22),**add(5)(q11.2)**,add(6)(q27),-15,0-3dmin[cp5]/72-82,idemx2,-1,-1,-11,-12,+0-2mar,0-16dmin[cp14]/46,XY[1] | 30 |
| 5q/7q-84 | F | 71 | Therapy related AML | 43-44,XX,?dic(3;14)(q29;p11.2),**add(5)(q31),-7**,der(9;17)(q10;q10),+mar[cp20] | 7 |
| 5q/7q-85 | M | 58 | AML-MRC | 44-45,XY,add(4)(q21),**add(5)(q13)**,add(6)(q15),**-7**,-10,add(10)(p13),-17,add(20)(p13),add(21)(q22),+1-2mar[cp20] | 11 |
| 5q/7q-86 | M | 70 | MDS/other myeloid malignancy | 42-43,XY,**del(5)(q32q31),-7**, 10,add(11)(q12),add(16)(q22),-17,-18,add(19)(p13.3),add(20)(q13.1),-22,+1-2mar[20] | 12 |
| 5q/7q-87 | M | 59 | De novo AML | 44,XY,**der(5;17)(p10;q10),del(7)(q22)**,del(8)(p21),del(9)(q13q22),add(12)(p11.2),-15,-20,+mar[9]/45,idem,+del(8)(p21)[6]/46,XY[5] | 10 |
| 5q/7q-88 | M | 58 | De novo AML | 44,XY,der(1;16)(q10;p10),**der(5)t(1;5)(p22;q21),add(7)(q11.2),**inv(12)(q13q15),del(13)(q12q22),add(13)(q32),der(18)t(1;18)(p22;p11.2),del(20)(q11.2q13.1),-22[9]/44,XY,der(1;16)(q10;p10),der(5)t(1;5)(p22;q21),-6,-7,add(12)(p13),+16,-18,del(20)(q11.2q13.1),-22,+r,+mar[9]/44,XY,der(1;16)(q10;p10),der(5)t(1;5)(p22;q21),-7,del(13)(q12q22),-18,del(20)(q11.2q13.1),-22,+mar[2] | 13 |
| 5q/7q-89 | M | 80 | De novo AML | 45-46,XY,inv(2)(p11.2q13)?c,**del(5)(q13q33)**,del(6)(q25),**-7**,dic(12;?)(p11.2;?),add(17)(p11.2),add(21)(q22),+0-1mar[19]/46,XY,inv(2)(p11.2q13)?c[1] | 9 |
| 5q/7q-90 | M | 77 | Relapsed AML | 43-44,XY,**der(5;17)(p10;q10),add(7)(q11.2)**,-13,-17,add(19)(p13.3),+mar[cp4]/41-44,sl,dic(15;21)(p11.2;p11.2)[cp12]/ 43,sl,i(11)(q10)[2]/46,XY[1] | 10 |
| 5q/7q-91 | M | 87 | De novo AML | 44,XY,**del(5)(q31q35)**,-6,**add(7)(q11.2)**,idic(11)(p11.2),add(17)(p11.2),psu dic(19;6)(p13.1;p23),-20[8]/ 43,XY,del(5)(q31q35),-6,add(7)(q11.2),der(11;18)(q10;q10),add(17)(p11.2),psu dic(19;6)(p13.1;p23),-20[7]/,46,XY[5] | 9 |
| 5q/7q-92 | F | 65 | De novo AML | 43-44,XX,add(1)(q21),-2,add(2)(q35),del(3)(p11),-4,**add(5)(q11.2),-7**,add(12)(p11.2),-14,+16,add(16)(p13.3),-17,add(17)(p11.2),-18,add(19)(q13.1),add(19)(q13.3),-20,add(21)(q22),+1-2r,+2-3mar[cp18]/46,XX[2] | 23 |
| 5q/7q-93 | M | 29 | De novo AML | 46-48,add(X)(p22.3),-Y,**-5**,add(10)(q26),-11,+13,+add(14)(q32),+1-2r,+mar[cp14]/46,XY[6] **FISH confirms 5q and 7q del** | 10 |
| 5q/7q-94 | F | 63 | Therapy related | 45,XX,**der(5;7)(p10;p10)**,add(12)(p11.2),add(17)(p11.2)[5]/45,sl,del(6)(q11)[13]/46,XX[2] | 5 |
| 5q/7q-95 | M | 71 | De novo AML | 46,XY,add(3)(q21),der(12)t(3;12)(q26;p13)[3]/39-45,sl,add(1)(q21),-add(3),**-5,-7,**add(7)(q32),+8,add(11)(q23),-12,-16,-21,-20,+22,+0-2mar[cp17] | 17 |
| 5q/7q-96 | F | 74 | De novo AML | 46-51,XX,-3,**dic(5;13)(q11.2;p11.2),del(7)(q22q34)**,der(10)t(3;10)(p11;q24),add(12)(p11.2),+2-7r,+0-2mar[cp20] | 16 |
| 5q/7q-97 | M | 73 | MDS/other myeloid malignancy | 43-44,XY,-1,-4,der(4)t(4;15)(q31.1;q15),**-5,-5,add(7)(q11.2)**,add(11)(q13),add(11)(q23),add(17)(q21),+1-3mar[cp11]/46,XY[9] | 13 |
| 5q/7q-98 | F | 70 | MDS/other myeloid malignancy | 46,XX,**add(5)(q11.2)**,add(6)(p12),**-7**,add(14)(q32),+r?(6)[10]/45,idem,+6,-add(6), der(7)t(7;12)(q22;p11.2)[3],add(12)(p11.2)[5],-r?(6)[cp10] | 11 |
| 5q/7q-99 | F | 64 | AML-MRC | 44,XX,der(2)t(2;3)(q37;p21),-3,**-7**,+i(8)(q10),-18[19]/46,XX[1] **FISH confirms 5q and 7 del** | 6 |
| 5q/7q-100 | M | 70 | AML-MRC | 44,XY,-2,**add(5)(q22),-7**,-13,del(17)(p11.2),add(21)(q22),+mar[20] | 7 |
| 5q/7q-101 | M | 89 | De novo AML | 41-44,XY,add(3)(p13),**der(3)t(3;5)(p21;q13),-5,der(5)t(5;12)(q13;q13),**-6,add(6)(q13),del(6)(p23),add(7)(p13),-11,add(11)(q13),-12,add(12)(p13),-13,del(13)(q12q22),add(14(p11.2),add(17)(p11.2),add(17)(q23),-18,-20,-21,-22,+0-4mar[cp20] **FISH confirms 5q and 7q del** | 27 |
| 5q/7q-102 | M | 61 | De novo AML | **Chromosomes fail. FISH confirms 5q and 7q del** | N/A |
| 5q/7q-103 | M | 58 | AML-MRC | 46-47,XY,del(3)(p21p23),**del(5)(q15q33),**-6,**del(7)(q22q34),**del(12)(p11.2p13),add(16)(q12),-18,del(20)(q11.2q13.1),+21,add(22)(q13),idic(22;22)(p11.2;p11.2)add(22)(q13),+2-3mar[cp20] | 14 |

Patient ID, sex, gender, morphologic diagnosis, conventional chromosome result and complexity based on chromosome results. ND: not done. While we aim to identify 20 metaphase cells, an * in the karyotype column indicates less than 20 metaphase cells were identified. To obtain the complexity #, all karyotype abnormalities were tallied if they fit one of the criteria described in the International System for Human Cytogenomic Nomenclature (ISCN) 2020*: aneusomy (1), deletion (1), duplication (1), balanced translocation (1), inversion (1), ring chromosome (1), unbalanced translocation (2), additional material on chromosome (1), each marker chromosome (1), each ring chromosome (1) and loss of chromosome Y (1).

*International Standing Committee on Human Cytogenomic Nomenclature, McGowan-Jordan J, Hastings RJ, Moore S. ISCN 2020: an international system for human cytogenomic nomenclature (2020). Basel ; Hartford: Karger; 2020.
