# Supplementary material for: Myeloid malignancies with 5q and 7q deletions are associated with extreme genomic complexity, biallelic TP53 variants, and very poor prognosis
Source: Blood Cancer J. 2021 Feb 8;11(2):18. doi: 10.1038/s41408-021-00416-4 (PMC7873204; doi:10.1038/s41408-021-00416-4)
Supplement: Supplementary file 4 — Table S3 [file 41408_2021_416_MOESM4_ESM.docx]

**Table S3: Copy number (CN) gains, losses and SVs in each subtype.**

| **Cohort** | **Number of cases** | **Genomic Abnormality** | | | | | | | | |
| --- | --- | --- | --- | --- | --- | --- | --- | --- | --- | --- |
|  |  | **Gains** | | | **Losses** | | | **SVs** | | |
|  |  | (Mean±s.d) | Median | *P* -value | (Mean±s.d) | Median | *P* -value | (Mean±s.d) | Median | *P*-value |
| **NK** | 37 | 2.56±1.67 | 2.0 | <0.001 | 4.32±2.24 | 5.0 | <0.001 | 3.86±2.25 | 4.0 | <0.001 |
| **7q del** | 7 | 3.42±2.14 | 2.0 |  | 7.00±2.16 | 8.0 |  | 6.71±3.30 | 6.0 |  |
| **5q del** | 10 | 9.80±7.31 | 11.5 |  | 16.70±12.31 | 14.5 |  | 31.60±32.21 | 17.5 |  |
| **5q/7q del** | 15 | 16.46±10.47 | 14.0 |  | 25.00±12.60 | 24.0 |  | 69.33±45.03 | 60.0 |  |

Kruskal-Wallis Test was conducted to examine the differences in CN gains, CN losses and SVs between subtypes.
