# Supplementary material for: Myeloid malignancies with 5q and 7q deletions are associated with extreme genomic complexity, biallelic TP53 variants, and very poor prognosis
Source: Blood Cancer J. 2021 Feb 8;11(2):18. doi: 10.1038/s41408-021-00416-4 (PMC7873204; doi:10.1038/s41408-021-00416-4)
Supplement: Supplementary file 5 — Table S4 [file 41408_2021_416_MOESM5_ESM.docx]

**Table S4: Copy Number Burden (CNB) in each subtype.**

| **Case ID** | **CNB** | **CNLB** | **CNGB** | **CNB** | **CNLB** | **CNGB** |
| --- | --- | --- | --- | --- | --- | --- |
|  | **Including 5q/7q** | | | **Excluding 5q/7q** | | |
| NK-1 | 0.00% | 0.00% | 0.00% | 0.00% | 0.00% | 0.00% |
| NK-2 | 4.23% | 1.16% | 3.07% | 4.57% | 1.26% | 3.31% |
| NK-3 | 0.01% | 0.01% | 0.00% | 0.01% | 0.01% | 0.00% |
| NK-4 | 0.02% | 0.01% | 0.01% | 0.02% | 0.01% | 0.01% |
| NK-5 | 0.02% | 0.00% | 0.02% | 0.02% | 0.00% | 0.02% |
| NK-6 | 0.01% | 0.01% | 0.00% | 0.01% | 0.01% | 0.00% |
| NK-7 | 0.12% | 0.12% | 0.00% | 0.10% | 0.10% | 0.00% |
| NK-8 | 0.00% | 0.00% | 0.00% | 0.00% | 0.00% | 0.00% |
| NK-9 | 0.04% | 0.03% | 0.01% | 0.04% | 0.03% | 0.01% |
| NK-10 | 0.01% | 0.00% | 0.01% | 0.01% | 0.00% | 0.01% |
| NK-11 | 0.06% | 0.01% | 0.05% | 0.06% | 0.01% | 0.05% |
| NK-12 | 0.07% | 0.03% | 0.03% | 0.01% | 0.01% | 0.00% |
| NK-13 | 0.07% | 0.01% | 0.06% | 0.08% | 0.01% | 0.06% |
| NK-14 | 0.03% | 0.02% | 0.01% | 0.03% | 0.02% | 0.01% |
| NK-15 | 0.12% | 0.03% | 0.09% | 0.10% | 0.03% | 0.07% |
| NK-16 | 0.05% | 0.01% | 0.03% | 0.03% | 0.01% | 0.02% |
| NK-17 | 0.69% | 0.52% | 0.17% | 0.27% | 0.16% | 0.11% |
| NK-18 | 0.03% | 0.00% | 0.03% | 0.03% | 0.00% | 0.03% |
| NK-19 | 0.08% | 0.06% | 0.02% | 0.09% | 0.06% | 0.02% |
| NK-20 | 0.15% | 0.05% | 0.10% | 0.16% | 0.05% | 0.11% |
| NK-21 | 0.05% | 0.03% | 0.01% | 0.05% | 0.03% | 0.01% |
| NK-22 | 0.08% | 0.05% | 0.04% | 0.09% | 0.05% | 0.04% |
| NK-23 | 0.06% | 0.04% | 0.02% | 0.06% | 0.04% | 0.02% |
| NK-24 | 0.07% | 0.03% | 0.03% | 0.07% | 0.03% | 0.03% |
| NK-25 | 0.09% | 0.09% | 0.00% | 0.10% | 0.10% | 0.00% |
| NK-26 | 0.03% | 0.03% | 0.00% | 0.03% | 0.03% | 0.00% |
| NK-27 | 0.06% | 0.01% | 0.05% | 0.06% | 0.01% | 0.05% |
| NK-28 | 0.17% | 0.14% | 0.03% | 0.18% | 0.15% | 0.03% |
| NK-29 | 0.01% | 0.01% | 0.00% | 0.01% | 0.01% | 0.00% |
| NK-30 | 0.03% | 0.01% | 0.02% | 0.03% | 0.01% | 0.02% |
| NK-31 | 0.06% | 0.03% | 0.02% | 0.06% | 0.05% | 0.02% |
| NK-32 | 0.03% | 0.03% | 0.00% | 0.03% | 0.03% | 0.00% |
| NK-33 | 0.11% | 0.10% | 0.01% | 0.10% | 0.09% | 0.01% |
| NK-34 | 0.06% | 0.05% | 0.01% | 0.06% | 0.05% | 0.01% |
| NK-35 | 0.05% | 0.02% | 0.04% | 0.03% | 0.02% | 0.02% |
| NK-36 | 0.02% | 0.01% | 0.01% | 0.02% | 0.01% | 0.01% |
| NK-37 | 0.05% | 0.02% | 0.02% | 0.05% | 0.02% | 0.02% |
| Median NK | 0.05% | 0.03% | 0.02% | 0.05% | 0.02% | 0.02% |
| 7q-53 | 8.14% | 8.13% | 0.01% | 5.09% | 5.08% | 0.01% |
| 7q-54 | 6.03% | 6.02% | 0.01% | 2.76% | 2.75% | 0.01% |
| 7q-55 | 7.54% | 4.28% | 3.26% | 4.52% | 1.01% | 3.52% |
| 7q-56 | 1.82% | 1.82% | 0.00% | 0.11% | 0.10% | 0.01% |
| 7q-57 | 10.62% | 5.56% | 5.06% | 7.76% | 2.26% | 5.50% |
| 7q-58 | 5.57% | 5.56% | 0.01% | 2.25% | 2.24% | 0.01% |
| 7q-59 | 3.89% | 2.81% | 1.08% | 1.33% | 0.16% | 1.17% |
| Median 7q del | 6.03% | 5.56% | 0.01% | 2.76% | 2.24% | 0.01% |
| 5q-65 | 4.86% | 3.92% | 0.94% | 3.83% | 2.81% | 1.03% |
| 5q-66 | 8.81% | 7.09% | 1.72% | 6.70% | 4.83% | 1.87% |
| 5q-67 | 13.80% | 7.36% | 6.46% | 11.59% | 4.58% | 7.01% |
| 5q-68 | 57.77% | 3.07% | 54.70% | 57.80% | 0.01% | 57.79% |
| 5q-69 | 10.92% | 10.24% | 0.69% | 10.39% | 9.66% | 0.74% |
| 5q-70 | 1.81% | 1.81% | 0.00% | 0.22% | 0.22% | 0.00% |
| 5q-71 | 0.56% | 0.55% | 0.01% | 0.03% | 0.02% | 0.01% |
| 5q-72 | 19.83% | 16.01% | 3.83% | 17.64% | 13.50% | 4.15% |
| 5q-73 | 9.20% | 5.14% | 4.07% | 6.91% | 2.52% | 4.38% |
| 5q-74 | 21.38% | 16.50% | 4.87% | 20.32% | 15.03% | 5.29% |
| Median 5q del | 10.06% | 6.12% | 2.77% | 8.65% | 3.69% | 3.01% |
| 5q/7q-84 | 13.74% | 10.43% | 3.31% | 9.28% | 5.67% | 3.61% |
| 5q/7q-85 | 16.20% | 15.51% | 0.69% | 9.77% | 9.02% | 0.74% |
| 5q/7q-86 | 14.25% | 13.53% | 0.71% | 10.68% | 9.91% | 0.77% |
| 5q/7q-87 | 19.45% | 12.68% | 6.76% | 13.83% | 6.95% | 6.87% |
| 5q/7q-88 | 21.95% | 16.53% | 5.42% | 17.57% | 11.86% | 5.72% |
| 5q/7q-89 | 8.49% | 6.27% | 2.23% | 2.84% | 0.42% | 2.42% |
| 5q/7q-90 | 18.93% | 14.20% | 4.74% | 12.78% | 6.05% | 6.73% |
| 5q/7q-91 | 15.19% | 9.80% | 5.39% | 11.66% | 5.12% | 6.54% |
| 5q/7q-92 | 22.47% | 17.65% | 4.82% | 17.46% | 12.25% | 5.21% |
| 5q/7q-93 | 16.77% | 9.72% | 7.05% | 11.66% | 5.11% | 6.54% |
| 5q/7q-94 | 13.20% | 12.86% | 0.34% | 8.22% | 7.86% | 0.36% |
| 5q/7q-95 | 37.30% | 30.85% | 6.45% | 35.13% | 28.06% | 7.08% |
| 5q/7q-96 | 11.08% | 10.16% | 0.92% | 4.93% | 3.92% | 1.01% |
| 5q/7q-97 | 11.79% | 9.69% | 2.09% | 5.99% | 3.81% | 2.18% |
| 5q/7q-98 | 5.90% | 5.88% | 0.01% | 0.00% | 0.00% | 0.00% |
| Median 5q/7q del | 15.19% | 12.68% | 3.31% | 10.68% | 6.05% | 3.61% |

The percent of the autosomal genome at an abnormal copy number were calculated and stratified into the percent observed as losses and gains.  These calculations were also performed excluding the long arms of chromosomes 5 and 7 to eliminate bias caused by inclusion of large deleted regions in the original calculation. CNB: copy number burden, CNLB: copy number loss burden, CNGB: copy number gain burden.
