# Supplementary material for: Myeloid malignancies with 5q and 7q deletions are associated with extreme genomic complexity, biallelic TP53 variants, and very poor prognosis
Source: Blood Cancer J. 2021 Feb 8;11(2):18. doi: 10.1038/s41408-021-00416-4 (PMC7873204; doi:10.1038/s41408-021-00416-4)
Supplement: Supplementary file 6 — Table S5 [file 41408_2021_416_MOESM6_ESM.docx]

**Table S5:** Univariate and multivariate Cox regression analyses of cytogenetic risk factors for overall survival.

| **Cytogenetic risk factors**  **(Cases = 95)** | **Univariate**  **Model** | | | | **Multivariate**  **Model** | | | |
| --- | --- | --- | --- | --- | --- | --- | --- | --- |
|  | **Risk Ratio** | **CI (95%)** | | **P-value** | **Risk Ratio** | **CI (95%)** | | **P-value** |
| **NK** | 0.458 | 0.279 | 0.751 | 0.002 | 0.633 | 0.357 | 1.122 | 0.117 |
| **7q del** | 0.679 | 0.324 | 1.424 | 0.306 |  |  |  |  |
| **5q del** | 1.613 | 0.876 | 2.969 | 0.124 |  |  |  |  |
| **5q/7q del** | 3.390 | 1.941 | 5.920 | <.0001 | 2.576 | 1.360 | 4.878 | 0.003 |

Only significant cytogenetic risk factors in univariate analysis were included in multivariate analysis. Abbreviations: CI, confidence interval
