# Supplementary material for: Myeloid malignancies with 5q and 7q deletions are associated with extreme genomic complexity, biallelic TP53 variants, and very poor prognosis
Source: Blood Cancer J. 2021 Feb 8;11(2):18. doi: 10.1038/s41408-021-00416-4 (PMC7873204; doi:10.1038/s41408-021-00416-4)
Supplement: Supplementary file 7 — Figure S1 [file 41408_2021_416_MOESM7_ESM.pptx]

## Slide 1
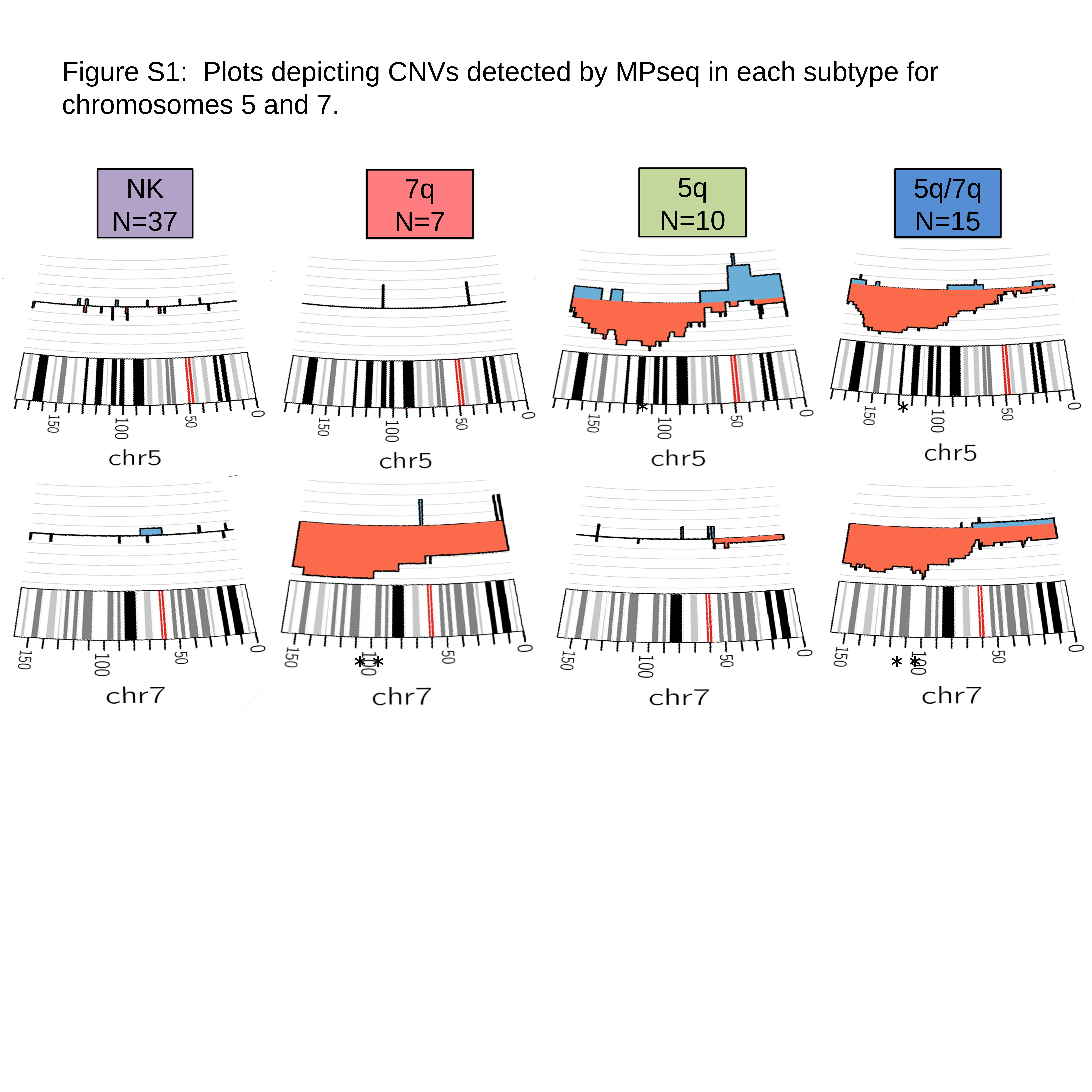

Figure S1: Plots depicting CNVs detected by MPseq in each subtype for chromosomes 5 and 7.
5q
N=10
5q/7q
N=15
NK
N=37
7q
N=7
*
*
**
**
