# Supplementary material for: Myeloid malignancies with 5q and 7q deletions are associated with extreme genomic complexity, biallelic TP53 variants, and very poor prognosis
Source: Blood Cancer J. 2021 Feb 8;11(2):18. doi: 10.1038/s41408-021-00416-4 (PMC7873204; doi:10.1038/s41408-021-00416-4)
Supplement: Supplementary file 8 — Figure S2 [file 41408_2021_416_MOESM8_ESM.pptx]

## Slide 1
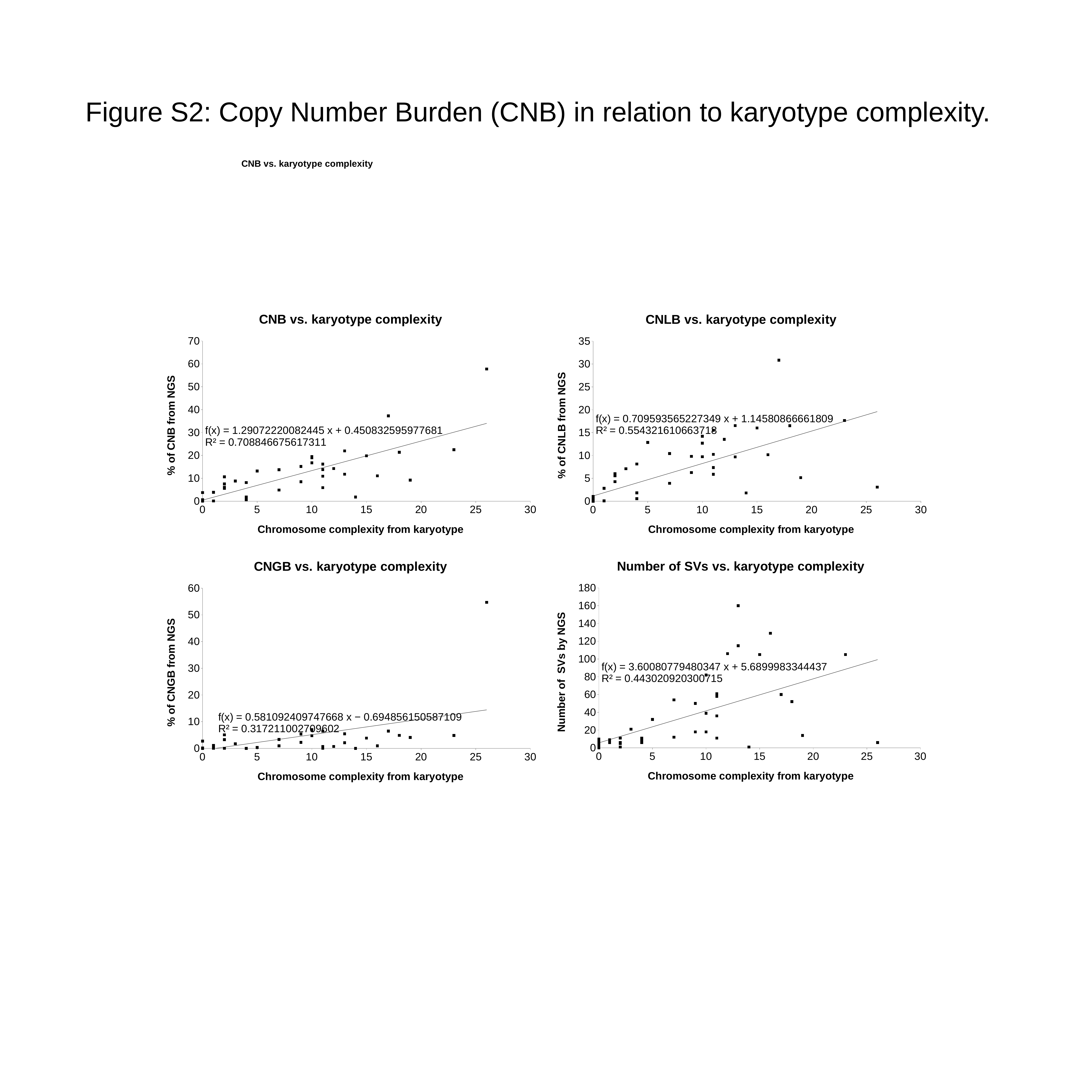

Figure S2: Copy Number Burden (CNB) in relation to karyotype complexity.
### Chart: CNB vs. karyotype complexity
| Category |
|---|
### Chart: CNB vs. karyotype complexity
| Category | |
|---|---|
### Chart: CNLB vs. karyotype complexity
| Category | |
|---|---|
### Chart: Number of SVs vs. karyotype complexity
| Category | |
|---|---|
### Chart: CNGB vs. karyotype complexity
| Category | |
|---|---|
