# Supplementary material for: Myeloid malignancies with 5q and 7q deletions are associated with extreme genomic complexity, biallelic TP53 variants, and very poor prognosis
Source: Blood Cancer J. 2021 Feb 8;11(2):18. doi: 10.1038/s41408-021-00416-4 (PMC7873204; doi:10.1038/s41408-021-00416-4)
Supplement: Supplementary file 9 — Figure S3 [file 41408_2021_416_MOESM9_ESM.pptx]

## Slide 1
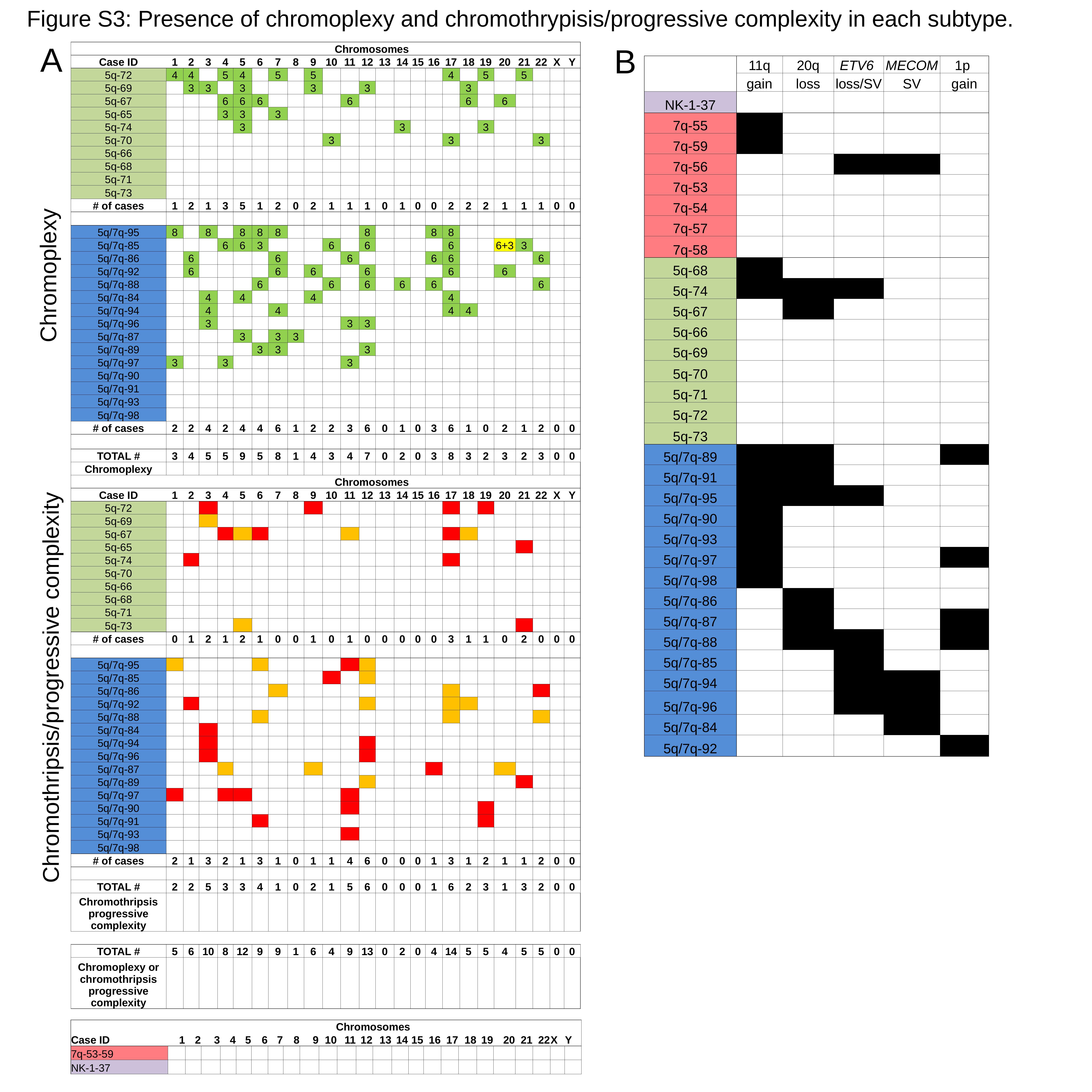

Figure S3: Presence of chromoplexy and chromothrypisis/progressive complexity in each subtype.
A
B
| | Chromosomes | | | | | | | | | | | | | | | | | | | | | | | |
| --- | --- | --- | --- | --- | --- | --- | --- | --- | --- | --- | --- | --- | --- | --- | --- | --- | --- | --- | --- | --- | --- | --- | --- | --- |
| Case ID | 1 | 2 | 3 | 4 | 5 | 6 | 7 | 8 | 9 | 10 | 11 | 12 | 13 | 14 | 15 | 16 | 17 | 18 | 19 | 20 | 21 | 22 | X | Y |
| 5q-72 | 4 | 4 | | 5 | 4 | | 5 | | 5 | | | | | | | | 4 | | 5 | | 5 | | | |
| 5q-69 | | 3 | 3 | | 3 | | | | 3 | | | 3 | | | | | | 3 | | | | | | |
| 5q-67 | | | | 6 | 6 | 6 | | | | | 6 | | | | | | | 6 | | 6 | | | | |
| 5q-65 | | | | 3 | 3 | | 3 | | | | | | | | | | | | | | | | | |
| 5q-74 | | | | | 3 | | | | | | | | | 3 | | | | | 3 | | | | | |
| 5q-70 | | | | | | | | | | 3 | | | | | | | 3 | | | | | 3 | | |
| 5q-66 | | | | | | | | | | | | | | | | | | | | | | | | |
| 5q-68 | | | | | | | | | | | | | | | | | | | | | | | | |
| 5q-71 | | | | | | | | | | | | | | | | | | | | | | | | |
| 5q-73 | | | | | | | | | | | | | | | | | | | | | | | | |
| # of cases | 1 | 2 | 1 | 3 | 5 | 1 | 2 | 0 | 2 | 1 | 1 | 1 | 0 | 1 | 0 | 0 | 2 | 2 | 2 | 1 | 1 | 1 | 0 | 0 |
| | | | | | | | | | | | | | | | | | | | | | | | | |
| 5q/7q-95 | 8 | | 8 | | 8 | 8 | 8 | | | | | 8 | | | | 8 | 8 | | | | | | | |
| 5q/7q-85 | | | | 6 | 6 | 3 | | | | 6 | | 6 | | | | | 6 | | | 6+3 | 3 | | | |
| 5q/7q-86 | | 6 | | | | | 6 | | | | 6 | | | | | 6 | 6 | | | | | 6 | | |
| 5q/7q-92 | | 6 | | | | | 6 | | 6 | | | 6 | | | | | 6 | | | 6 | | | | |
| 5q/7q-88 | | | | | | 6 | | | | 6 | | 6 | | 6 | | 6 | | | | | | 6 | | |
| 5q/7q-84 | | | 4 | | 4 | | | | 4 | | | | | | | | 4 | | | | | | | |
| 5q/7q-94 | | | 4 | | | | 4 | | | | | | | | | | 4 | 4 | | | | | | |
| 5q/7q-96 | | | 3 | | | | | | | | 3 | 3 | | | | | | | | | | | | |
| 5q/7q-87 | | | | | 3 | | 3 | 3 | | | | | | | | | | | | | | | | |
| 5q/7q-89 | | | | | | 3 | 3 | | | | | 3 | | | | | | | | | | | | |
| 5q/7q-97 | 3 | | | 3 | | | | | | | 3 | | | | | | | | | | | | | |
| 5q/7q-90 | | | | | | | | | | | | | | | | | | | | | | | | |
| 5q/7q-91 | | | | | | | | | | | | | | | | | | | | | | | | |
| 5q/7q-93 | | | | | | | | | | | | | | | | | | | | | | | | |
| 5q/7q-98 | | | | | | | | | | | | | | | | | | | | | | | | |
| # of cases | 2 | 2 | 4 | 2 | 4 | 4 | 6 | 1 | 2 | 2 | 3 | 6 | 0 | 1 | 0 | 3 | 6 | 1 | 0 | 2 | 1 | 2 | 0 | 0 |
| | | | | | | | | | | | | | | | | | | | | | | | | |
| TOTAL # | 3 | 4 | 5 | 5 | 9 | 5 | 8 | 1 | 4 | 3 | 4 | 7 | 0 | 2 | 0 | 3 | 8 | 3 | 2 | 3 | 2 | 3 | 0 | 0 |
| Chromoplexy | | | | | | | | | | | | | | | | | | | | | | | | |
| | Chromosomes | | | | | | | | | | | | | | | | | | | | | | | |
| Case ID | 1 | 2 | 3 | 4 | 5 | 6 | 7 | 8 | 9 | 10 | 11 | 12 | 13 | 14 | 15 | 16 | 17 | 18 | 19 | 20 | 21 | 22 | X | Y |
| 5q-72 | | | | | | | | | | | | | | | | | | | | | | | | |
| 5q-69 | | | | | | | | | | | | | | | | | | | | | | | | |
| 5q-67 | | | | | | | | | | | | | | | | | | | | | | | | |
| 5q-65 | | | | | | | | | | | | | | | | | | | | | | | | |
| 5q-74 | | | | | | | | | | | | | | | | | | | | | | | | |
| 5q-70 | | | | | | | | | | | | | | | | | | | | | | | | |
| 5q-66 | | | | | | | | | | | | | | | | | | | | | | | | |
| 5q-68 | | | | | | | | | | | | | | | | | | | | | | | | |
| 5q-71 | | | | | | | | | | | | | | | | | | | | | | | | |
| 5q-73 | | | | | | | | | | | | | | | | | | | | | | | | |
| # of cases | 0 | 1 | 2 | 1 | 2 | 1 | 0 | 0 | 1 | 0 | 1 | 0 | 0 | 0 | 0 | 0 | 3 | 1 | 1 | 0 | 2 | 0 | 0 | 0 |
| | | | | | | | | | | | | | | | | | | | | | | | | |
| 5q/7q-95 | | | | | | | | | | | | | | | | | | | | | | | | |
| 5q/7q-85 | | | | | | | | | | | | | | | | | | | | | | | | |
| 5q/7q-86 | | | | | | | | | | | | | | | | | | | | | | | | |
| 5q/7q-92 | | | | | | | | | | | | | | | | | | | | | | | | |
| 5q/7q-88 | | | | | | | | | | | | | | | | | | | | | | | | |
| 5q/7q-84 | | | | | | | | | | | | | | | | | | | | | | | | |
| 5q/7q-94 | | | | | | | | | | | | | | | | | | | | | | | | |
| 5q/7q-96 | | | | | | | | | | | | | | | | | | | | | | | | |
| 5q/7q-87 | | | | | | | | | | | | | | | | | | | | | | | | |
| 5q/7q-89 | | | | | | | | | | | | | | | | | | | | | | | | |
| 5q/7q-97 | | | | | | | | | | | | | | | | | | | | | | | | |
| 5q/7q-90 | | | | | | | | | | | | | | | | | | | | | | | | |
| 5q/7q-91 | | | | | | | | | | | | | | | | | | | | | | | | |
| 5q/7q-93 | | | | | | | | | | | | | | | | | | | | | | | | |
| 5q/7q-98 | | | | | | | | | | | | | | | | | | | | | | | | |
| # of cases | 2 | 1 | 3 | 2 | 1 | 3 | 1 | 0 | 1 | 1 | 4 | 6 | 0 | 0 | 0 | 1 | 3 | 1 | 2 | 1 | 1 | 2 | 0 | 0 |
| | | | | | | | | | | | | | | | | | | | | | | | | |
| TOTAL # | 2 | 2 | 5 | 3 | 3 | 4 | 1 | 0 | 2 | 1 | 5 | 6 | 0 | 0 | 0 | 1 | 6 | 2 | 3 | 1 | 3 | 2 | 0 | 0 |
| Chromothripsis progressive complexity | | | | | | | | | | | | | | | | | | | | | | | | |
| | | | | | | | | | | | | | | | | | | | | | | | | |
| TOTAL # | 5 | 6 | 10 | 8 | 12 | 9 | 9 | 1 | 6 | 4 | 9 | 13 | 0 | 2 | 0 | 4 | 14 | 5 | 5 | 4 | 5 | 5 | 0 | 0 |
| Chromoplexy or chromothripsis progressive complexity | | | | | | | | | | | | | | | | | | | | | | | | |
| | 11q | 20q | ETV6 | MECOM | 1p |
| --- | --- | --- | --- | --- | --- |
| | gain | loss | loss/SV | SV | gain |
| NK-1-37 | | | | | |
| 7q-55 | 1 | | | | |
| 7q-59 | 1 | | | | |
| 7q-56 | | | 1 | 1 | |
| 7q-53 | | | | | |
| 7q-54 | | | | | |
| 7q-57 | | | | | |
| 7q-58 | | | | | |
| 5q-68 | 1 | | | | |
| 5q-74 | 1 | 1 | 1 | | |
| 5q-67 | | 1 | | | |
| 5q-66 | | | | | |
| 5q-69 | | | | | |
| 5q-70 | | | | | |
| 5q-71 | | | | | |
| 5q-72 | | | | | |
| 5q-73 | | | | | |
| 5q/7q-89 | 1 | 1 | | | 1 |
| 5q/7q-91 | 1 | 1 | | | |
| 5q/7q-95 | 1 | 1 | 1 | | |
| 5q/7q-90 | 1 | | | | |
| 5q/7q-93 | 1 | | | | |
| 5q/7q-97 | 1 | | | | 1 |
| 5q/7q-98 | 1 | | | | |
| 5q/7q-86 | | 1 | | | |
| 5q/7q-87 | | 1 | | | 1 |
| 5q/7q-88 | | 1 | 1 | | 1 |
| 5q/7q-85 | | | 1 | | |
| 5q/7q-94 | | | 1 | 1 | |
| 5q/7q-96 | | | 1 | 1 | |
| 5q/7q-84 | | | | 1 | |
| 5q/7q-92 | | | | | 1 |
Chromoplexy
Chromothripsis/progressive complexity
| | Chromosomes | | | | | | | | | | | | | | | | | | | | | | | |
| --- | --- | --- | --- | --- | --- | --- | --- | --- | --- | --- | --- | --- | --- | --- | --- | --- | --- | --- | --- | --- | --- | --- | --- | --- |
| Case ID | 1 | 2 | 3 | 4 | 5 | 6 | 7 | 8 | 9 | 10 | 11 | 12 | 13 | 14 | 15 | 16 | 17 | 18 | 19 | 20 | 21 | 22 | X | Y |
| 7q-53-59 | | | | | | | | | | | | | | | | | | | | | | | | |
| NK-1-37 | | | | | | | | | | | | | | | | | | | | | | | | |
