# Supplementary material for: Myeloid malignancies with 5q and 7q deletions are associated with extreme genomic complexity, biallelic TP53 variants, and very poor prognosis
Source: Blood Cancer J. 2021 Feb 8;11(2):18. doi: 10.1038/s41408-021-00416-4 (PMC7873204; doi:10.1038/s41408-021-00416-4)
Supplement: Supplementary file 10 — Figure S4 [file 41408_2021_416_MOESM10_ESM.pptx]

## Slide 1
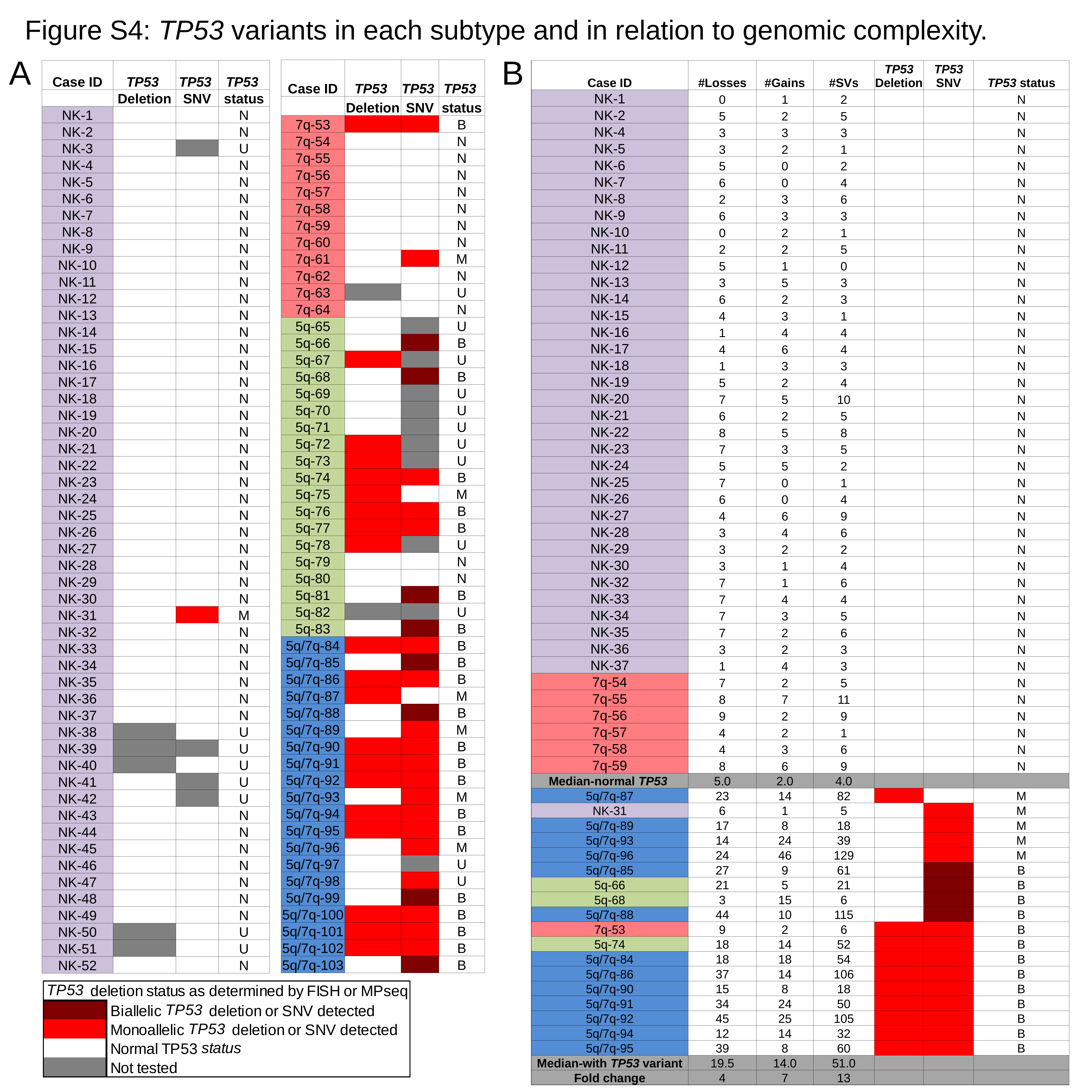

Figure S4: TP53 variants in each subtype and in relation to genomic complexity.
A
B
| Case ID | TP53 | TP53 | TP53 |
| --- | --- | --- | --- |
| | Deletion | SNV | status |
| 7q-53 | 1 | 1 | B |
| 7q-54 | | | N |
| 7q-55 | | | N |
| 7q-56 | | | N |
| 7q-57 | | | N |
| 7q-58 | | | N |
| 7q-59 | | | N |
| 7q-60 | | | N |
| 7q-61 | | 1 | M |
| 7q-62 | | | N |
| 7q-63 | | | U |
| 7q-64 | | | N |
| 5q-65 | | | U |
| 5q-66 | | 1 | B |
| 5q-67 | 1 | | U |
| 5q-68 | | 1 | B |
| 5q-69 | | | U |
| 5q-70 | | | U |
| 5q-71 | | | U |
| 5q-72 | 1 | | U |
| 5q-73 | 1 | | U |
| 5q-74 | 1 | 1 | B |
| 5q-75 | 1 | | M |
| 5q-76 | 1 | 1 | B |
| 5q-77 | 1 | 1 | B |
| 5q-78 | 1 | | U |
| 5q-79 | | | N |
| 5q-80 | | | N |
| 5q-81 | | 1 | B |
| 5q-82 | | | U |
| 5q-83 | | 1 | B |
| 5q/7q-84 | 1 | 1 | B |
| 5q/7q-85 | | 1 | B |
| 5q/7q-86 | 1 | 1 | B |
| 5q/7q-87 | 1 | | M |
| 5q/7q-88 | | 1 | B |
| 5q/7q-89 | | 1 | M |
| 5q/7q-90 | 1 | 1 | B |
| 5q/7q-91 | 1 | 1 | B |
| 5q/7q-92 | 1 | 1 | B |
| 5q/7q-93 | | 1 | M |
| 5q/7q-94 | 1 | 1 | B |
| 5q/7q-95 | 1 | 1 | B |
| 5q/7q-96 | | 1 | M |
| 5q/7q-97 | | | U |
| 5q/7q-98 | | 1 | U |
| 5q/7q-99 | | 1 | B |
| 5q/7q-100 | 1 | 1 | B |
| 5q/7q-101 | 1 | 1 | B |
| 5q/7q-102 | 1 | 1 | B |
| 5q/7q-103 | | 1 | B |
| Case ID | TP53 | TP53 | TP53 |
| --- | --- | --- | --- |
| | Deletion | SNV | status |
| NK-1 | | | N |
| NK-2 | | | N |
| NK-3 | | | U |
| NK-4 | | | N |
| NK-5 | | | N |
| NK-6 | | | N |
| NK-7 | | | N |
| NK-8 | | | N |
| NK-9 | | | N |
| NK-10 | | | N |
| NK-11 | | | N |
| NK-12 | | | N |
| NK-13 | | | N |
| NK-14 | | | N |
| NK-15 | | | N |
| NK-16 | | | N |
| NK-17 | | | N |
| NK-18 | | | N |
| NK-19 | | | N |
| NK-20 | | | N |
| NK-21 | | | N |
| NK-22 | | | N |
| NK-23 | | | N |
| NK-24 | | | N |
| NK-25 | | | N |
| NK-26 | | | N |
| NK-27 | | | N |
| NK-28 | | | N |
| NK-29 | | | N |
| NK-30 | | | N |
| NK-31 | | 1 | M |
| NK-32 | | | N |
| NK-33 | | | N |
| NK-34 | | | N |
| NK-35 | | | N |
| NK-36 | | | N |
| NK-37 | | | N |
| NK-38 | | | U |
| NK-39 | | | U |
| NK-40 | | | U |
| NK-41 | | | U |
| NK-42 | | | U |
| NK-43 | | | N |
| NK-44 | | | N |
| NK-45 | | | N |
| NK-46 | | | N |
| NK-47 | | | N |
| NK-48 | | | N |
| NK-49 | | | N |
| NK-50 | | | U |
| NK-51 | | | U |
| NK-52 | | | N |
| Case ID | #Losses | #Gains | #SVs | TP53 Deletion | TP53 SNV | TP53 status |
| --- | --- | --- | --- | --- | --- | --- |
| NK-1 | 0 | 1 | 2 | | | N |
| NK-2 | 5 | 2 | 5 | | | N |
| NK-4 | 3 | 3 | 3 | | | N |
| NK-5 | 3 | 2 | 1 | | | N |
| NK-6 | 5 | 0 | 2 | | | N |
| NK-7 | 6 | 0 | 4 | | | N |
| NK-8 | 2 | 3 | 6 | | | N |
| NK-9 | 6 | 3 | 3 | | | N |
| NK-10 | 0 | 2 | 1 | | | N |
| NK-11 | 2 | 2 | 5 | | | N |
| NK-12 | 5 | 1 | 0 | | | N |
| NK-13 | 3 | 5 | 3 | | | N |
| NK-14 | 6 | 2 | 3 | | | N |
| NK-15 | 4 | 3 | 1 | | | N |
| NK-16 | 1 | 4 | 4 | | | N |
| NK-17 | 4 | 6 | 4 | | | N |
| NK-18 | 1 | 3 | 3 | | | N |
| NK-19 | 5 | 2 | 4 | | | N |
| NK-20 | 7 | 5 | 10 | | | N |
| NK-21 | 6 | 2 | 5 | | | N |
| NK-22 | 8 | 5 | 8 | | | N |
| NK-23 | 7 | 3 | 5 | | | N |
| NK-24 | 5 | 5 | 2 | | | N |
| NK-25 | 7 | 0 | 1 | | | N |
| NK-26 | 6 | 0 | 4 | | | N |
| NK-27 | 4 | 6 | 9 | | | N |
| NK-28 | 3 | 4 | 6 | | | N |
| NK-29 | 3 | 2 | 2 | | | N |
| NK-30 | 3 | 1 | 4 | | | N |
| NK-32 | 7 | 1 | 6 | | | N |
| NK-33 | 7 | 4 | 4 | | | N |
| NK-34 | 7 | 3 | 5 | | | N |
| NK-35 | 7 | 2 | 6 | | | N |
| NK-36 | 3 | 2 | 3 | | | N |
| NK-37 | 1 | 4 | 3 | | | N |
| 7q-54 | 7 | 2 | 5 | | | N |
| 7q-55 | 8 | 7 | 11 | | | N |
| 7q-56 | 9 | 2 | 9 | | | N |
| 7q-57 | 4 | 2 | 1 | | | N |
| 7q-58 | 4 | 3 | 6 | | | N |
| 7q-59 | 8 | 6 | 9 | | | N |
| Median-normal TP53 | 5.0 | 2.0 | 4.0 | | | |
| 5q/7q-87 | 23 | 14 | 82 | 1 | | M |
| NK-31 | 6 | 1 | 5 | | 1 | M |
| 5q/7q-89 | 17 | 8 | 18 | | 1 | M |
| 5q/7q-93 | 14 | 24 | 39 | | 1 | M |
| 5q/7q-96 | 24 | 46 | 129 | | 1 | M |
| 5q/7q-85 | 27 | 9 | 61 | | 1 | B |
| 5q-66 | 21 | 5 | 21 | | 1 | B |
| 5q-68 | 3 | 15 | 6 | | 1 | B |
| 5q/7q-88 | 44 | 10 | 115 | | 1 | B |
| 7q-53 | 9 | 2 | 6 | 1 | 1 | B |
| 5q-74 | 18 | 14 | 52 | 1 | 1 | B |
| 5q/7q-84 | 18 | 18 | 54 | 1 | 1 | B |
| 5q/7q-86 | 37 | 14 | 106 | 1 | 1 | B |
| 5q/7q-90 | 15 | 8 | 18 | 1 | 1 | B |
| 5q/7q-91 | 34 | 24 | 50 | 1 | 1 | B |
| 5q/7q-92 | 45 | 25 | 105 | 1 | 1 | B |
| 5q/7q-94 | 12 | 14 | 32 | 1 | 1 | B |
| 5q/7q-95 | 39 | 8 | 60 | 1 | 1 | B |
| Median-with TP53 variant | 19.5 | 14.0 | 51.0 | | | |
| Fold change | 4 | 7 | 13 | | | |
