# Supplementary material for: Myeloid malignancies with 5q and 7q deletions are associated with extreme genomic complexity, biallelic TP53 variants, and very poor prognosis
Source: Blood Cancer J. 2021 Feb 8;11(2):18. doi: 10.1038/s41408-021-00416-4 (PMC7873204; doi:10.1038/s41408-021-00416-4)
Supplement: Supplementary file 11 — Figure S5 [file 41408_2021_416_MOESM11_ESM.pptx]

## Slide 1
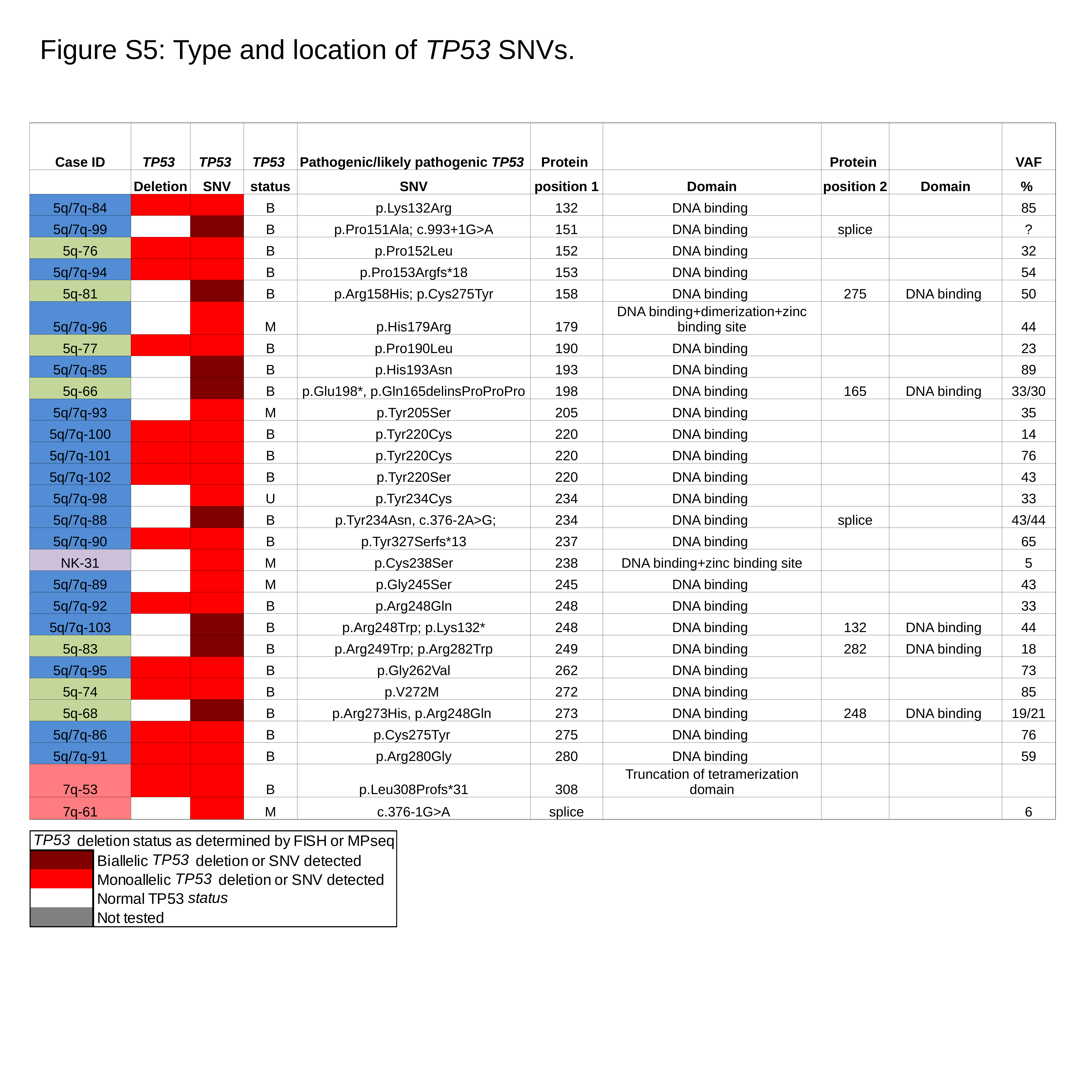

Figure S5: Type and location of TP53 SNVs.
| Case ID | TP53 | TP53 | TP53 | Pathogenic/likely pathogenic TP53 | Protein | | Protein | | VAF |
| --- | --- | --- | --- | --- | --- | --- | --- | --- | --- |
| | Deletion | SNV | status | SNV | position 1 | Domain | position 2 | Domain | % |
| 5q/7q-84 | 1 | 1 | B | p.Lys132Arg | 132 | DNA binding | | | 85 |
| 5q/7q-99 | | 1 | B | p.Pro151Ala; c.993+1G>A | 151 | DNA binding | splice | | ? |
| 5q-76 | 1 | 1 | B | p.Pro152Leu | 152 | DNA binding | | | 32 |
| 5q/7q-94 | 1 | 1 | B | p.Pro153Argfs\*18 | 153 | DNA binding | | | 54 |
| 5q-81 | | 1 | B | p.Arg158His; p.Cys275Tyr | 158 | DNA binding | 275 | DNA binding | 50 |
| 5q/7q-96 | | 1 | M | p.His179Arg | 179 | DNA binding+dimerization+zinc binding site | | | 44 |
| 5q-77 | 1 | 1 | B | p.Pro190Leu | 190 | DNA binding | | | 23 |
| 5q/7q-85 | | 1 | B | p.His193Asn | 193 | DNA binding | | | 89 |
| 5q-66 | | 1 | B | p.Glu198\*, p.Gln165delinsProProPro | 198 | DNA binding | 165 | DNA binding | 33/30 |
| 5q/7q-93 | | 1 | M | p.Tyr205Ser | 205 | DNA binding | | | 35 |
| 5q/7q-100 | 1 | 1 | B | p.Tyr220Cys | 220 | DNA binding | | | 14 |
| 5q/7q-101 | 1 | 1 | B | p.Tyr220Cys | 220 | DNA binding | | | 76 |
| 5q/7q-102 | 1 | 1 | B | p.Tyr220Ser | 220 | DNA binding | | | 43 |
| 5q/7q-98 | | 1 | U | p.Tyr234Cys | 234 | DNA binding | | | 33 |
| 5q/7q-88 | | 1 | B | p.Tyr234Asn, c.376-2A>G; | 234 | DNA binding | splice | | 43/44 |
| 5q/7q-90 | 1 | 1 | B | p.Tyr327Serfs\*13 | 237 | DNA binding | | | 65 |
| NK-31 | | 1 | M | p.Cys238Ser | 238 | DNA binding+zinc binding site | | | 5 |
| 5q/7q-89 | | 1 | M | p.Gly245Ser | 245 | DNA binding | | | 43 |
| 5q/7q-92 | 1 | 1 | B | p.Arg248Gln | 248 | DNA binding | | | 33 |
| 5q/7q-103 | | 1 | B | p.Arg248Trp; p.Lys132\* | 248 | DNA binding | 132 | DNA binding | 44 |
| 5q-83 | | 1 | B | p.Arg249Trp; p.Arg282Trp | 249 | DNA binding | 282 | DNA binding | 18 |
| 5q/7q-95 | 1 | 1 | B | p.Gly262Val | 262 | DNA binding | | | 73 |
| 5q-74 | 1 | 1 | B | p.V272M | 272 | DNA binding | | | 85 |
| 5q-68 | | 1 | B | p.Arg273His, p.Arg248Gln | 273 | DNA binding | 248 | DNA binding | 19/21 |
| 5q/7q-86 | 1 | 1 | B | p.Cys275Tyr | 275 | DNA binding | | | 76 |
| 5q/7q-91 | 1 | 1 | B | p.Arg280Gly | 280 | DNA binding | | | 59 |
| 7q-53 | 1 | 1 | B | p.Leu308Profs\*31 | 308 | Truncation of tetramerization domain | | | |
| 7q-61 | | 1 | M | c.376-1G>A | splice | | | | 6 |
