# Supplementary material for: Myeloid malignancies with 5q and 7q deletions are associated with extreme genomic complexity, biallelic TP53 variants, and very poor prognosis
Source: Blood Cancer J. 2021 Feb 8;11(2):18. doi: 10.1038/s41408-021-00416-4 (PMC7873204; doi:10.1038/s41408-021-00416-4)
Supplement: Supplementary file 12 — Figure S6 [file 41408_2021_416_MOESM12_ESM.pptx]

## Slide 1
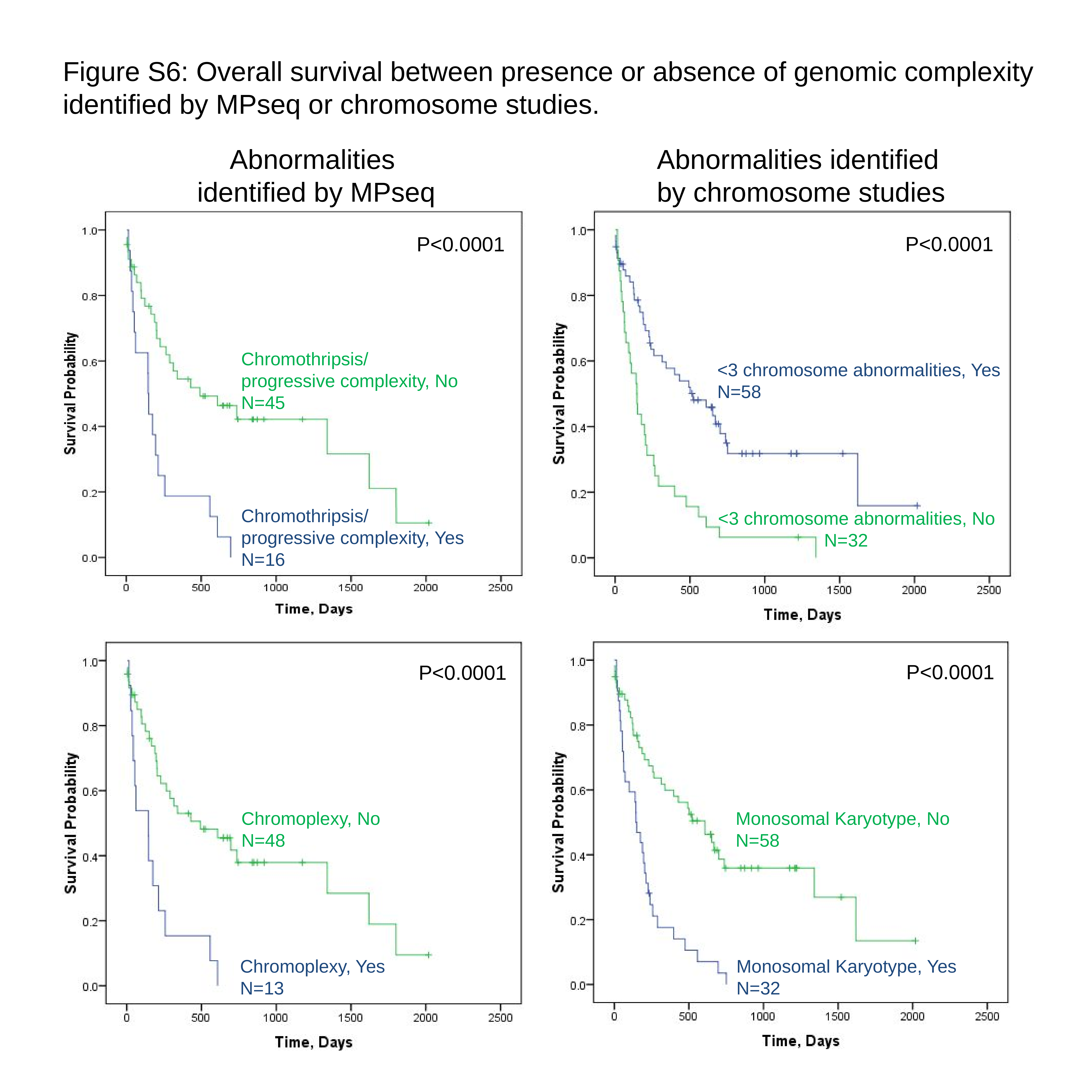

Figure S6: Overall survival between presence or absence of genomic complexity identified by MPseq or chromosome studies.
Abnormalities
identified by MPseq
Abnormalities identified
by chromosome studies
P<0.0001
P<0.0001
Chromothripsis/
progressive complexity, No
N=45
<3 chromosome abnormalities, Yes
N=58
Chromothripsis/
progressive complexity, Yes
N=16
<3 chromosome abnormalities, No
 N=32
P<0.0001
P<0.0001
Chromoplexy, No
N=48
Monosomal Karyotype, No
N=58
Monosomal Karyotype, Yes
N=32
Chromoplexy, Yes
N=13
